# FBXL8 Stabilizes IκBα and Negatively Regulated NF-κB Activation to Suppress Pancreatic Cancer Progression

**DOI:** 10.7150/ijbs.122689

**Published:** 2026-01-08

**Authors:** Chunming Li, Kui Fu, Feifan Wu, Zhihao Fan, Yongpeng Gu, Chaohua Zhang, Qin Lang, Zhu Zhu, Xiong Ding, Jianping Gong, Junhua Gong

**Affiliations:** 1 Department of Hepatobiliary Surgery, The Second Affiliated Hospital of Chongqing Medical University, Chongqing 400010, China.; 2 Department of Cardiothoracic, Thyroid and Breast Surgery, The Shapingba Hospital, Chongqing University (People's Hospital of Shapingba District, Chongqing), Chongqing 400030, China.; 3 Department of Hepatobiliary and Pancreatic Surgery, The Third Affiliated Hospital of Chongqing Medical University, Chongqing 401120, China.; 4 Department of Urinary Nephropathy Center, Second Hospital Affiliated to Chongqing Medical University, Chongqing 400010, China.; 5 Department of Clinical Nutrition, Chongqing Red Cross Hospital (People's Hospital of Jiangbei District), Chongqing 401100, China.

**Keywords:** FBXL8, IκBα, NF-κB, ubiquitination, pancreatic cancer

## Abstract

The dysregulation of ubiquitin-proteasome system (UPS) causes various diseases including cancer. The NF-κB signaling pathway, a critical regulator of inflammation and cell survival, is constitutively activated in pancreatic cancer (PC), but the role of UPS in its regulation is incompletely elucidated. Here, we found that E3 ubiquitin ligase FBXL8 is downregulated in PC tissues, and associated with poor patient prognosis. Functional experiments show that FBXL8 suppresses PC cells proliferation, migration, and invasion both *in vitro* and *in vivo*. Mechanistically, FBXL8 binds to dephosphorylated IκBα (S32/S36) and mediates K63-linked polyubiquitination at the K38 site of IκBα, thereby stabilizing IκBα and inhibiting NF-κB p65 nuclear translocation. Meanwhile, p65 upregulates the transcription factor YY1, which transcriptionally represses FBXL8 expression, thereby forming a FBXL8-NF-κB feedforward regulatory loop. In conclusion, this study reveals that FBXL8 suppresses PC progression by stabilizing IκBα through non-degradative ubiquitination, and its downregulation via the NF-κB-YY1 axis promotes oncogenic progression. The FBXL8-IκBα-NF-κB pathway represents a promising novel therapeutic target for PC.

## Introduction

Pancreatic cancer (PC), particularly pancreatic ductal adenocarcinoma (PDAC), ranks among the most aggressive and treatment-resistant malignancies, with a 5-year survival rate below 10%^1^. Its insidious onset, rapid progression, and profound resistance to conventional therapies contribute to its dismal prognosis. Over 80% of cases present with locally advanced or metastatic disease, losing the opportunity for surgical resection at the time of diagnosis. Despite the development of targeted therapies like KRAS G12C inhibitors (e.g. sotorasib) and PARP inhibitors, the clinical benefits remain limited due to the rapid emergence of drug resistance^2^, ^3^. A comprehensive elucidation of the molecular mechanisms underlying pancreatic carcinogenesis and progression is critically required to inform the rational design of novel targeted therapeutic interventions.

NF-κB serves as crucial molecular bridges, converting extracellular signals into intracellular responses that regulate diverse cellular functions. In various tumors including PC, pro-inflammatory cytokines, DNA damage, and ROS drive constitutive NF-κB activation^4^-^7^. NF-κB dimers (primarily p50/p65) bind to κB promoter/enhancer elements to regulate transcription of target genes, including pro-inflammatory cytokines (TNF-α, IL-1β, IL-6), anti-apoptotic proteins (Bcl-2, Bcl-xL, XIAP), cell cycle regulators (Cyclin D1, c-Myc), and metastasis-promoting factors (MMP-9, VEGF)^8^-^12^. NF-κB also interacts with epigenetic modifiers like p300/CBP to facilitate histone acetylation and chromatin remodeling, while simultaneously influencing DNA methylation patterns at certain target loci^13^. Additionally, NF-κB signaling pathway exhibits extensive crosstalk with other oncogenic signaling pathways which frequently dysregulated in cancer (MAPK, JAK-STAT, Wnt/β-catenin), creating an integrated regulatory circuit that governs inflammation, cell survival, proliferation, and immune evasion^14^-^16^. This multilayered regulatory architecture establishes NF-κB as a master regulator orchestrating tumor progression and therapeutic resistance in diverse malignancies. Thereby, unraveling the sophisticated regulation of NF-κB signaling promises to reveal novel intervention points for disrupting oncogenic processes in cancer therapeutics.

Post-translation modifications (PTMs), including phosphorylation and ubiquitination, are essential for NF-κB signaling activation. IκB Kinase (IKK) complex or Casein Kinase 2 (CK2) phosphorylates IκBα, triggering β-transducin repeat-containing protein (β-TrCP)-mediated ubiquitination and degradation, which releases p50/p65 for nuclear translocation and activation^17^-^19^. However, the regulatory functions of non-degradative ubiquitination in NF-κB signaling remain poorly characterized.

Here, we report E3 ubiquitin ligase FBXL8 is downregulated and associated with poor prognosis in PC patients. Mechanistically, FBXL8 binds to dephosphorylated IκBα (S32/S36) and mediates its non-degradative polyubiquitination at K38, thereby stabilizing IκBα and suppressing PC progression. NF-κB p65 upregulates the expression of the transcription factor YY1, which directly binds to the FBXL8 promoter and transcriptionally represses FBXL8 expression. These findings reveal a FBXL8-NF-κB feedforward loop as a novel potential therapeutic target for human PC.

## Materials and Methods

### Patients and tissue specimens

Twenty pairs of tumor samples and paired adjacent non-tumor samples were collected from PC patients who underwent surgery in the Department of Hepatobiliary Surgery, the Second Affiliated Hospital of Chongqing Medical University, between October 2022 and October 2023. The diagnosis of all patients was confirmed by two independent pathologists. Additionally, 79 pairs of tissue microarrays from PC patients were purchased from Aifang Biotechnology (Changsha, China). This study was approved by the Ethics Committee of the Second Affiliated Hospital of Chongqing Medical University and complied with the Declaration of Helsinki. The informed consent was obtained from every patient.

### Cell culture, plasmid construction, transfection, and infection

The human embryonic kidney cell line HEK293T, human hepatocellular carcinoma cell line Huh7, human breast cancer cell line MCF-7, human lung adenocarcinoma cell line A549, normal pancreatic duct epithelial cell line HPDE6-C7, and human PC cell lines PANC-1 and CFPAC were purchased from the Cell Resource Center of the Shanghai Institute of Biological Sciences. HEK293T, Huh7, A549, MCF-7, and PANC-1 were cultured in Dulbecco's modified Eagle's medium (DMEM) supplemented with 10% fetal bovine serum (FBS). HPDE6-C7 were cultured in Roswell Park Memorial Institute 1640 (RPMI-1640) supplemented with 10% FBS. CFPAC were cultured in *Iscove's Modified Dulbecco's Medium* (IMDM) supplemented with 10% FBS.

Mammalian expression plasmids for Flag-, HA-, or His-tagged FBXL8, IκBα, Ubiquitin (Ub), β-TrCP, YY1, and p65 were constructed by the standard molecular cloning method from cDNA templates. Different points mutants of IκBα (K21R, K22R, K38R, K47R, K67R, S32A, S36A, Y42F, S283A, S288A, Y289F, T291A, S293A, T296A, T299A, and Y305F) and Ub (K48R and K63R) were generated by site-directed mutagenesis. The small interference RNA (siRNA) was purchased from GenePharma Co., Ltd. (Shanghai, China). Plasmids and siRNAs were transfected into cells using Lipofectamine 2000 reagent (Invitrogen, USA) following the manufacturer's instructions. Cells were infected with lentivirus (LeapWal, China) containing core coding region (CDS) of FBXL8 or the short hairpin RNA (shRNA) targeting FBXL8 and ABL1 to establish stable cell lines. The siRNA and shRNA sequences are presented in Table S1 and S2.

### Western blot (WB)

Total protein was lysed with RIPA Lysis Buffer (P0013B, Beyotime, China) supplemented with 1% protease inhibitor cocktail (Selleck, USA) on ice for 20 min. The supernatant was collected after centrifugation at 12,000 g for 10 min at 4 ℃. BCA Protein Assay Kit (P0012, Beyotime, China) was used to measure the protein concentration. SDS-PAGE Sample Loading Buffer (P0015L, Beyotime, China) was added, and the solutions was boiled for 10 min. All protein samples were analyzed by SDS-PAGE and transferred to NC membranes (GE Healthcare, UK). After blocked with 5% skim milk for 1 h at room temperature, the membranes were incubated with primary antibodies at 4 °C overnight. Subsequently, corresponding secondary antibodies were applied for 1 h at room temperature. Finally, the enhanced chemiluminescence (ECL) detection reagent (KeyGEN, China) and gel imaging system (Bio-Rad, USA) were used for visualization. Image Lab™ 4.0 software (Bio-Rad, USA) was used to analysis blots. The antibodies are presented in Table S3.

### RNA extraction and quantitative real-time PCR (qRT-PCR)

Total RNA was extracted with Trizol (Takara, Japan). Reverse transcription of mRNA was performed using PrimeScript RT reagent kit (Takara, Japan). qRT-PCR was conducted with SYBR Green Fast qPCR Mix (Abclonal, China) on qRT-PCR instrument (Bio-Rad, USA). The primers are presented in Table S4.

### Chromatin immunoprecipitation (ChIP)

The ChIP assay was performed using the ChIP Assay Kit (P2078, Beyotime, China) according to the manufacturer's protocols. Briefly, cells were cross-linked with 1% formaldehyde for 10 min, and terminated with glycine. After ultrasonication, the supernatant was collected and incubated with Protein A/G agarose beads and primary antibodies overnight at 4 ℃. After that, DNA was purified, extracted, and qPCR was performed. The ChIP primers are presented in Table S5.

### Mouse xenograft tumor models

6-week-old male BALB/c-nu mice were purchased from GemPharmatech Co., Ltd. (Chengdu, China) and fed in the Experimental Animal Center of Chongqing Medical University. The mice were cared for in accordance with the Regulations for the Administration of Affairs Concerning Experimental Animals. Stable cell lines of PANC-1 (5×10^6^) were subcutaneously injected into the right axillary side of the mice. Tumor volume was calculated every four days after being visible by the formula: volume = (width^2^×length)/2. To evaluate the effect of NF-κB inhibitors on PANC-1 with FBXL8 dysregulation, when tumors reached a size of 50 mm^3^, the mice of shNC and shFBXL8 groups were both treated daily by oral gavage administration with vehicle or JSH-23 (20 mg/kg) for 20 days. At 34 days after implantation, the mice were sacrificed, and tumor tissues were collected for further experiments. To evaluate the synergistic effect of the NF-κB inhibitor JSH-23 combined with gemcitabine on FBXL8-dysregulated PANC-1 xenograft tumors, when tumors reached a size of 50 mm^3^, mice were treated daily with JSH-23 (20 mg/kg) via oral gavage and every other day with gemcitabine (14.25 mg/kg) via intraperitoneal injection for 20 days. At 34 days after implantation, the mice were sacrificed, and tumor tissues were collected for further experiments.

### Statistics

GraphPad Prism 9.0 software (San Diego, CA) was used to analyze data, which are presented as the mean ± SEM. Two-tailed unpaired t-test was used to compare the differences between independent samples, one-way analysis of variance (ANOVA) was used to compare the differences among multiple groups. Pearson's chi-square test was used to analysis the clinicopathological correlations. Kaplan‒Meier method was used to explore the overall survival (OS). Pearson's correlation test was used to investigate the expression correlation between FBXL8 and IκBα or FBXL8 and nuclear p65. P values < 0.05 were considered statistically significant.

The other materials and methods are presented in **Supplementary materials and methods.**

## Results

### FBXL8 is downregulated in PC and correlates with poor prognosis

**Figure 1:**
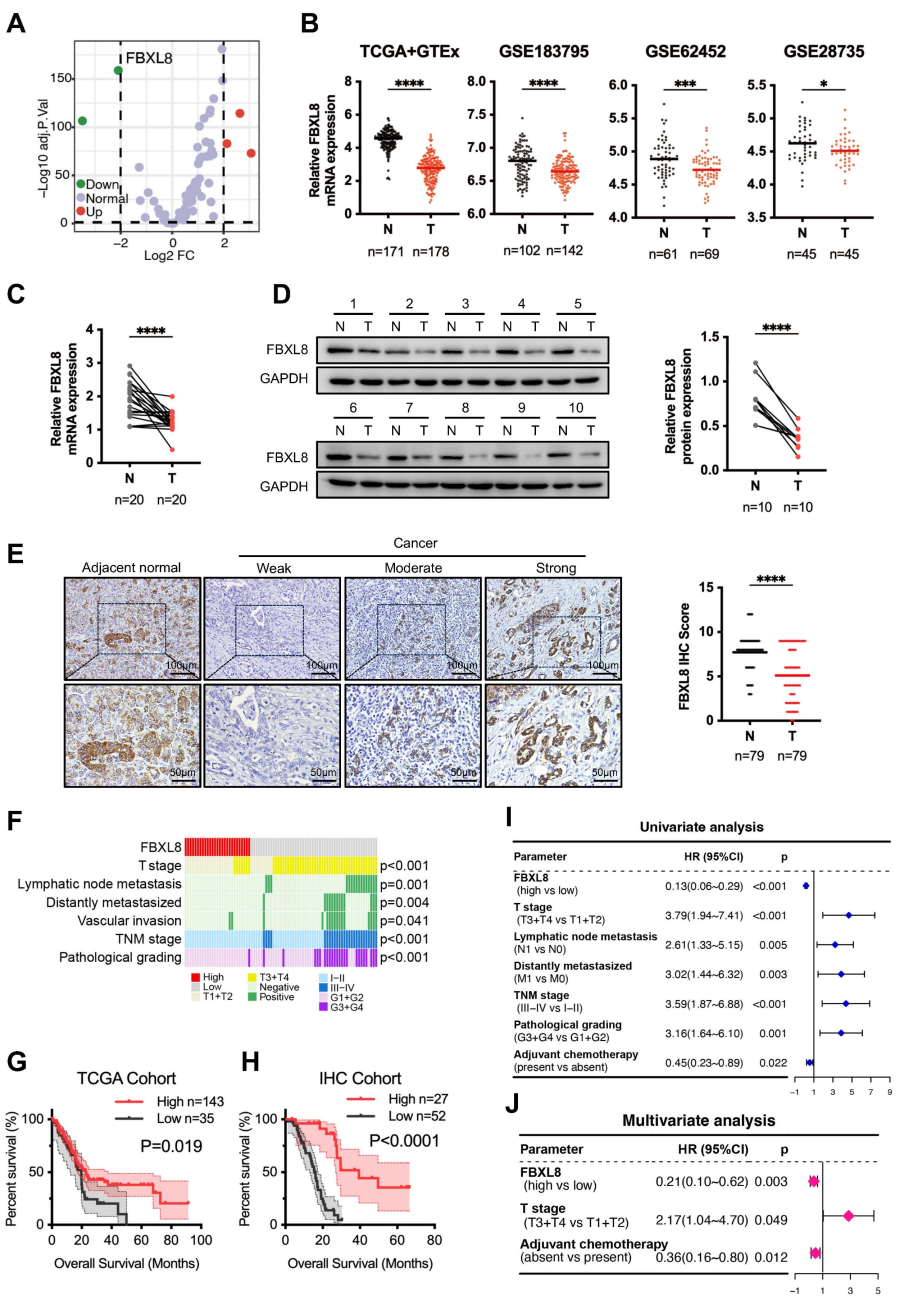
** Decreased FBXL8 expression correlates with poor prognosis in PC patients. (A)** Volcano plot of the expression of F-box family proteins comparing PC tumors with ANT in TCGA and GTEx database. **(B)** Relative FBXL8 mRNA levels in PC tumors (T) and ANT (N) from TCGA and GTEx, GSE183795, GSE62452, and GSE28735 databases. **(C)** Relative FBXL8 mRNA levels in human PC samples were determined by qRT-PCR. **(D)** Protein levels of FBXL8 in human PC samples were determined by WB. **(E)** Representative IHC staining images of FBXL8 in paired PC tumors (T) and ANT (N) (n=79). The IHC score was calculated by multiplying the percentage of positive cells and the intensity of staining. **(F)** Correlation heatmap between FBXL8 expression level of IHC cohort and clinical pathological features. **(G, H)** Decreased FBXL8 expression was positively associated with poor OS in PC patients of TCGA (G) and IHC (H) cohorts. **(I, J)** Univariate (I) and multivariate (J) analyses showing the risk factors associated with OS in the IHC cohort. Data are presented as the mean ± SEM; significance determined by Student's *t*-test (B-E). *p < 0.05, **p < 0.01, ***p < 0.001, ****p < 0.0001.

To identify F-box family proteins playing critical roles in PC progression, we analyzed the expression profiles of these proteins using PC data from The Cancer Genome Atlas (TCGA) and The Genotype-Tissue Expression (GTEx) databases. FBXL8 was found to be significantly downregulated in PC tissues compared to adjacent non-tumor tissues (ANT) (Fig. 1A, B). Consistently, analysis of the Gene Expression Omnibus (GEO) database also revealed reduced FBXL8 expression in PC tissues (Fig. 1B). In our center's PC patient specimens, both mRNA and protein levels of FBXL8 were lower in tumor tissues than in ANT (Fig. 1C, D). Immunohistochemistry (IHC) staining showed that FBXL8 was downregulated in 73.42% (58/79) of PC patients (Fig. 1E). Low FBXL8 expression was associated with higher T stage, lymphatic node metastasis rate, distant metastasis rate, potential vascular invasion, TNM stage, and pathological grading (Fig. 1F, Table S6). In both the TCGA cohort and IHC cohort, low FBXL8 expression correlated with shorter OS in PC patients (Fig. 1G, H). Univariate and multivariate Cox regression analyses confirmed that FBXL8 was an independent risk factor for PC prognosis (Fig. 1I, J, Table S7). These findings indicate that FBXL8 is downregulated in PC, correlates with tumor progression, and serves as an independent risk factor for PC prognosis.

### FBXL8 suppresses PC progression

**Figure 2:**
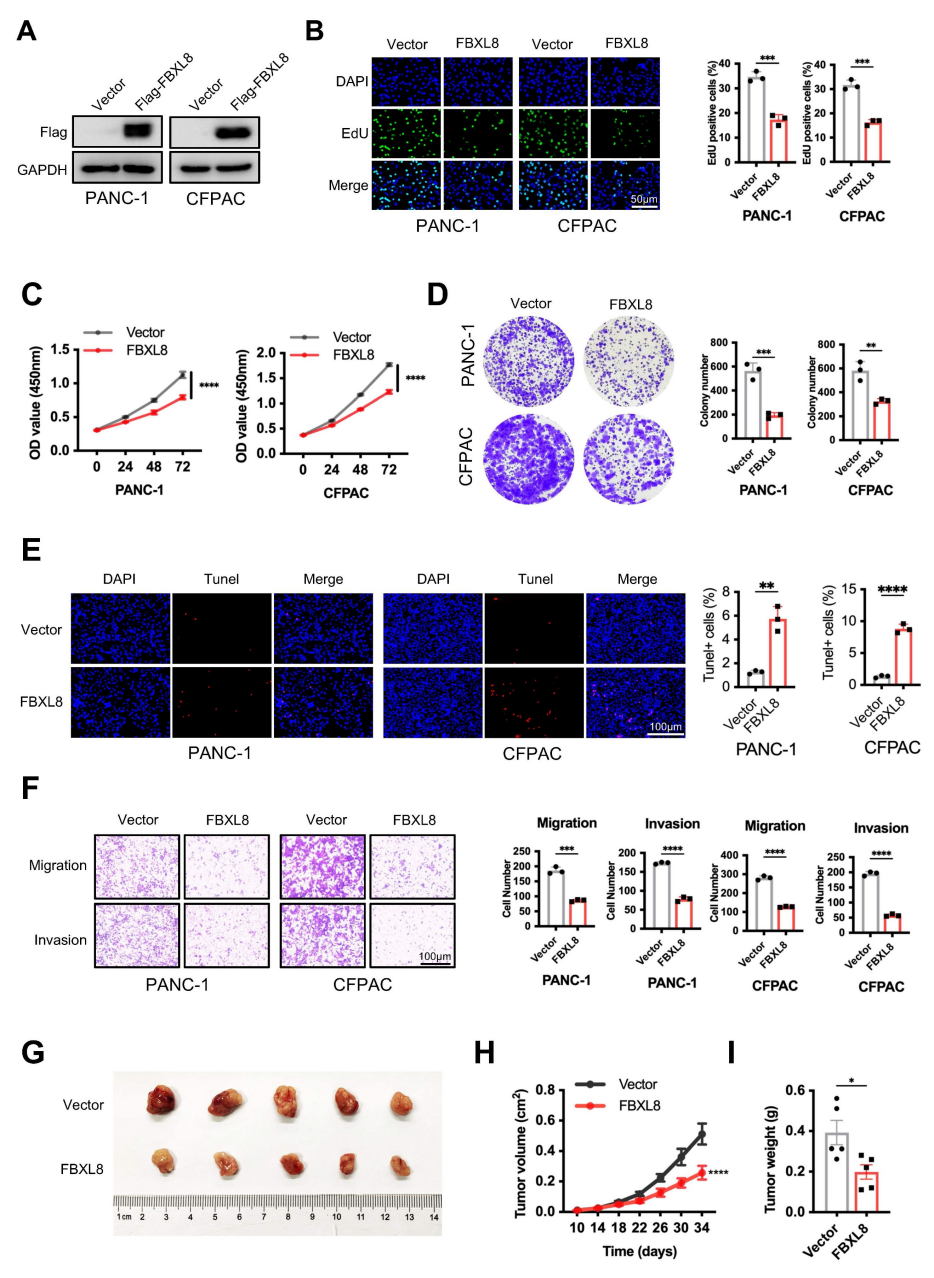
** FBXL8 inhibits PC growth and metastasis *in vitro* and *in vivo*. (A-F)** Flag-FBXL8 was constructed in PANC-1 and CFPAC cells. The efficiency of FBXL8 overexpression was detected by WB (A). Cell proliferation was measured by EDU (B), CCK-8 (C), and clone formation (D) assays. Cell apoptosis was measured using Tunel staining (E). Transwell assays measured cell migration and invasion ability (F). **(G-I)** Effects of FBXL8 overexpression on tumor growth in xenograft tumor model of PANC-1 cells. Macroscopic images were showed (G), and tumor size (H) and tumor weight (I) were determined. Data are presented as the mean ± SEM; significance determined by Student's unpaired *t*-test (B-F, H, I). *p < 0.05, **p < 0.01, ***p < 0.001, ****p < 0.0001.

To elucidate the specific role of FBXL8 in PC progression, we overexpressed FBXL8 in PC cells (Fig. 2A). FBXL8 overexpression significantly inhibited PC cells proliferation and colony formation, while promoting cell apoptosis (Fig. 2B-E). Transwell assays demonstrated that FBXL8 overexpression suppressed migration and invasion of PC cells (Fig. 2F). Furthermore, FBXL8 overexpression inhibited the growth of xenograft tumors *in vivo* (Fig. 2G-I). In contrast, FBXL8 knockdown significantly promoted PC cells proliferation and colony formation, accompanied by elevated the ability of cell migration and invasion (Fig. S1A-F). These data confirm that FBXL8 suppresses PC progression.

### FBXL8 binds to IκBα and enhances its stability

**Figure 3:**
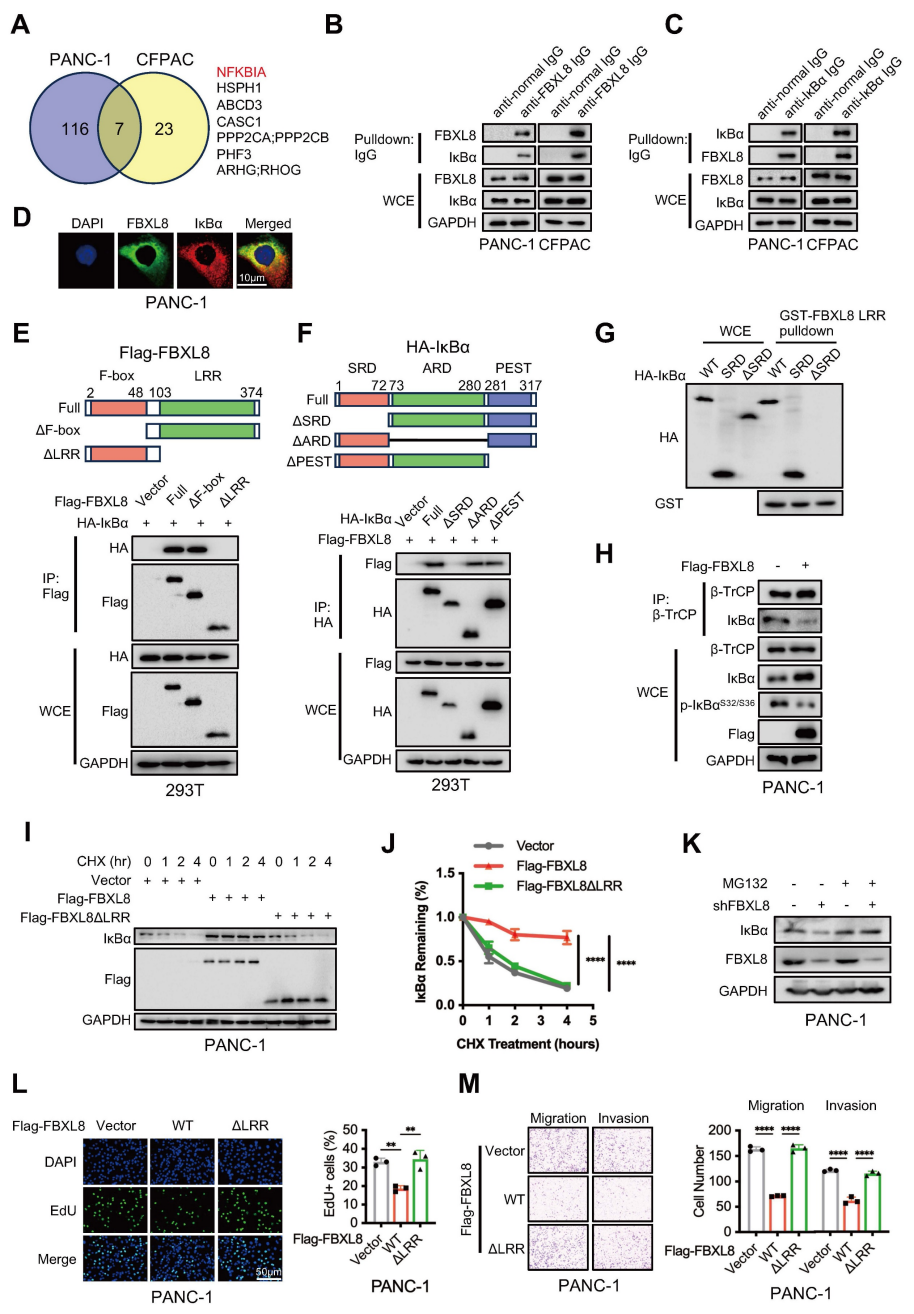
** FBXL8 directly interacts with IκBα and stabilizes it. (A)** Venn diagram showing the potential substrates of FBXL8 identified by co-IP and MS in PANC-1 and CFPAC cells. **(B, C)** The co-IP assays were performed using anti-normal immunoglobulin (IgG) or anti-FBXL8 antibody (B) and anti-normal IgG or anti-IκBα antibody (C) in PANC-1 and CFPAC cells. **(D)** Immunofluorescence staining showing the colocalization of endogenous FBXL8 (Green) and IκBα (Red) in PANC-1 cells. (**E**) 293T cells were co-transfected with different domains-deleted Flag-FBXL8 and HA-IκBα plasmids, then treated with MG132 (10 μM) for 4h. The co-IP assay was performed using anti-Flag antibody. (**F**) 293T cells were co-transfected with different domains-deleted HA-IκBα and Flag-FBXL8 plasmids for 48 h, then treated with MG132 (10 μM) for 4h. The co-IP assay was performed using anti-HA antibody. **(G)** 293T cells were transfected with WT, SRD, and ΔSRD HA-IκBα for 48 h, then lysed and incubated with recombinant GST-FBXL8 LRR protein, followed by GST pulldown and WB analysis. **(H)** PANC-1 cells were transfected with Vector or Flag-FBXL8 plasmid for 48 h. The co-IP assays were performed using anti-β-TrCP antibody. **(I)** PANC-1 cells were transfected with Vector, Flag-FBXL8 or Flag-FBXL8ΔLRR for 48 h, followed by Cycloheximide (CHX, 100 μg/mL) treatment for indicated times and the WB was used to detect the protein stability of IκBα. **(J)** Compared to 0 time point, the relative percentage of protein expression levels was quantified across different CHX treatment time points.** (K)** PANC-1 cells were transfected with shFBXL8 for 48 h and then treated with MG132 (10 μM) for 5 h. Cells were lysed and protein expression analyzed by WB. **(L, M)** PANC-1 cells were transfected with Vector, Flag-FBXL8 or Flag-FBXL8ΔLRR. Cell proliferation was measured using EDU assays (L). Transwell assays measured cell migration and invasion ability (M). Data are presented as the mean ± SEM; significance determined by one-way ANOVA with Tukey's multiple comparison (J, L, M). *p < 0.05, **p < 0.01, ***p < 0.001, ****p < 0.0001.

To further explore the specific mechanism by which FBXL8 influences PC progression, we performed MS to analyze proteins bound to FBXL8. NFKBIA (encoding IκBα) was a commonly identified protein in both PANC-1 and CFPAC cells with the highest number of peptides (Fig. 3A, Table S8, S9). Consistently, IκBα was also one of the predicted substrates of FBXL8 on UbiBrowser website (http://ubibrowser.bio-it.cn/ubibrowser_v3/) (Fig. S2A). Co-immunoprecipitation (co-IP) assays showed that both exogenous and endogenous FBXL8 interacted with IκBα (Fig. 3B, C, S2B, C). Immunofluorescence staining confirmed the cytoplasmic colocalization of FBXL8 and IκBα in PC cells (Fig. 3D). Plasmids encoding FBXL8 and IκBα with mutations in different functional domains were generated to explore specific binding regions. Co-IP experiments revealed that the leucine-rich repeat (LRR) domain of FBXL8 and the serin-rich domain (SRD) of IκBα were crucial for their interaction (Fig. 3E, F). To confirm the direct interaction between the LRR domain of FBXL8 and the SRD of IκBα, *in vitro* glutathione S-transferase (GST)-pulldown assays were performed. The results showed that GST-FBXL8 fusion protein containing the LRR domain interacted with IκBα or the SRD of IκBα, but not with IκBα with lacking the SRD (Fig. 3G). In addition, we utilized molecular docking to validate the structural basis for the FBXL8-IκBα interaction and delineated the specific amino acid residues potentially involved in this binding (Fig. S2D). To further confirm that the LRR domain of FBXL8 and the SRD domain of IκBα are critical regions mediating their endogenous binding, we found that overexpression FBXL8 LRR domain, but not FBXL8 LRR domain deletion mutation, could compete with endogenous full-length FBXL8 proteins for binding on IκBα (Fig. S2E, F). Similarly, overexpression IκBα SRD, but not IκBα SRD mutation, could compete with endogenous interaction of FBXL8 and IκBα (Fig. S2G, H). IKK complex phosphorylates IκBα at Ser32 and Ser36, triggering β-TrCP-mediated ubiquitination at Lys21 and Lys22 and subsequent degradation, which releases p50/p65 for nuclear translocation and activation^17^-^19^. Considering that the binding region of FBXL8 to IκBα includes these phosphorylation and ubiquitination sites, we explored whether this interaction disrupts the classical IκBα degradation pathway. Overexpression of FBXL8 reduced the phosphorylation of IκBα at Ser32 and Ser36, and the interaction between β-TrCP and IκBα, suggesting that FBXL8 could inhibit the classical IκBα degradation pathway by binding to the IκBα N-terminal region (Fig. 3H). Half-life assays showed that FBXL8 promoted IκBα stability, whereas LRR-deleted FBXL8 lost this stabilizing effect (Fig. 3I, J). In addition, the reduction of IκBα mediated by FBXL8 knockdown was abrogated by the proteasome inhibitor MG132, indicating that FBXL8 could reduce IκBα degradation through the ubiquitin-proteasome system (UPS) (Fig. 3K). Furthermore, LRR-deleted FBXL8 failed to inhibit PC cells proliferation, migration, and invasion (Fig. 3L, M, S3A, B). These results suggest that FBXL8 binds to IκBα and enhances its stability.

### FBXL8 mediates K63-linked ubiquitination of IκBα at Lys38

**Figure 4:**
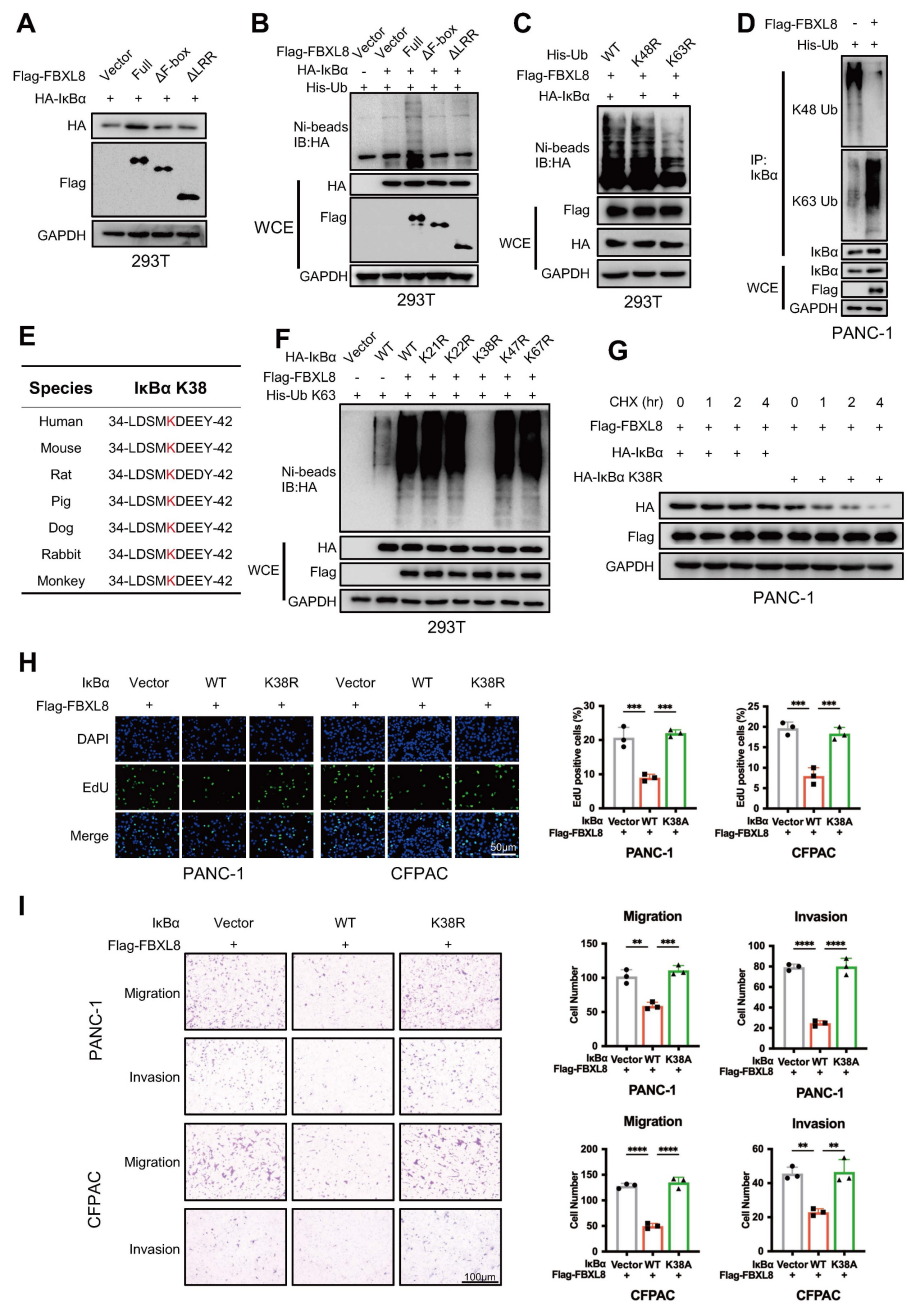
** FBXL8 promotes K63-linked ubiquitination of IκBα at K38 sites. (A)** 293T cells were co-transfected with different domains-deleted Flag-FBXL8 and HA-IκBα plasmids for 48 h, then the WB assay was performed to measure the protein levels. **(B)** 293T cells were co-transfected with different domains-deleted Flag-FBXL8, HA-IκBα, and His-Ub plasmids for 48 h. Cells were lysed with ubiquitination lysis buffer followed by pull-down using Ni-NTA beads, and precipitates were analyzed by WB. **(C)** 293T cells were co-transfected with WT or mutant His-Ub (K48R, K63R), Flag-FBXL8, and HA-IκBα plasmids for 48 h. Cells were lysed with ubiquitination lysis buffer followed by pull-down using Ni-NTA beads, and precipitates were analyzed by WB. **(D)** PANC-1 cells were transfected with Vector or Flag-FBXL8 and His-Ub plasmids for 48 h, then treated with MG132 (10 μM) for 4h. The co-IP assays were performed using anti-IκBα antibody. **(E)** Amino acid sequences of IκBα from the indicated species are highly conserved around the K38 sites. **(F)** 293T cells were co-transfected with WT or mutant HA-IκBα (K21R, K22R, K38R, K47R, K67R), Flag-FBXL8, and His-Ub K63 plasmids for 48 h. Cells were lysed with ubiquitination lysis buffer followed by pull-down using Ni-NTA beads, and precipitates were analyzed by WB. **(G)** PANC-1 cells were transfected with HA-IκBα or HA-IκBα K38R, and Flag-FBXL8 for 48 h, followed by Cycloheximide (CHX, 100 μg/mL) treatment for indicated times and the WB was used to detect the protein stability of HA-IκBα.** (H, I)** PANC-1 and CFPAC cells were transfected with Vector, WT or mutant IκBα (K38R) and Flag-FBXL8. Cell proliferation was measured using EDU assays (H). Transwell assays measured cell migration and invasion ability (I). Data are presented as the mean ± SEM; significance determined by one-way ANOVA with Tukey's multiple comparison (H, I). *p < 0.05, **p < 0.01, ***p < 0.001, ****p < 0.0001.

The F-box domain mediates binding to the SCF complex, and the LRR region mediates binding to the substrate IκBα. Full-length FBXL8 promoted IκBα stabilization via ubiquitination, whereas F-box or LRR-deleted mutants failed to ubiquitinate IκBα (Fig. 4A, B). To determine the type of IκBα ubiquitination mediated by FBXL8, *in vivo* ubiquitination assays were performed using Lys48- and Lys63-mutated Ub. Results showed that Lys63 mutation suppressed FBXL8-induced IκBα ubiquitination, indicating FBXL8 mediates K63-linked ubiquitination of IκBα (Fig. 4C). The endogenous K63 Ub modification of IκBα was significantly enhanced in PANC-1 cells with FBXL8 overexpression compared to control cells (Fig. 4D). Mass Spectrometry (MS) identified two potential ubiquitination sites (Lys38 and Lys47) of IκBα in FBXL8 overexpression PANC-1 and CFPAC cells (Table S10, S11). Combined with three previously reported sites (Lys21, Lys22, and Lys67)^20^, ^21^, we constructed mutated plasmids in which each of these five lysine residues was substituted with arginine (K→R). Only the IκBα K38R mutation abolished FBXL8-mediated ubiquitination, confirming that FBXL8 mediates K63-linked ubiquitination of IκBα at Lys38 (Fig. 4E, F). Half-life assays demonstrated that FBXL8 enhances the stability of exogenous wild-type (WT) IκBα, but not the IκBα K38R mutant (Fig. 4G). Functionally, WT IκBα suppressed PC cells proliferation, invasion, and migration, whereas the K38R mutant failed to exert these inhibitory effects (Fig. 4H, I). These results indicate that Lys38 is essential for FBXL8-mediated IκBα stabilization via polyubiquitination.

### IκBα phosphorylation at Y305 drives S32/S36 dephosphorylation, enabling subsequent FBXL8 binding

**Figure 5:**
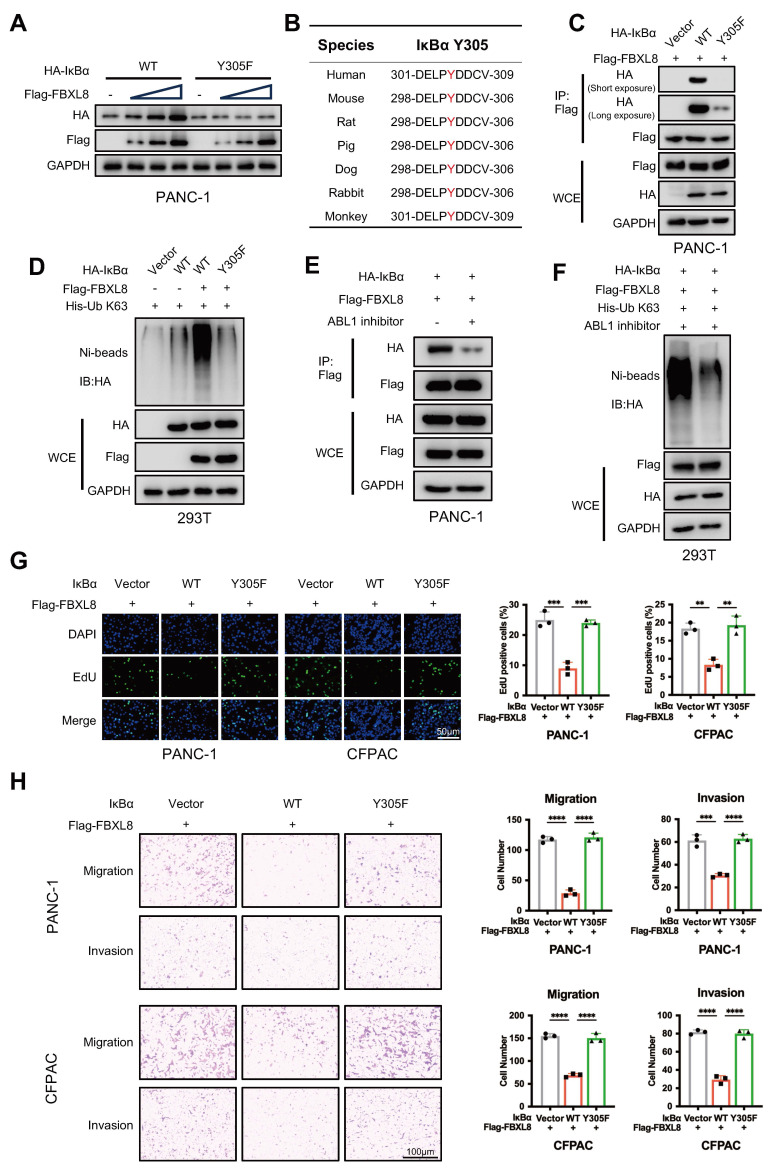
** IκBα phosphorylation at Y305 drives S32/S36 dephosphorylation, enabling subsequent FBXL8 binding. (A)** PANC-1 cells were transfected with WT or mutant HA-IκBα (Y305F) and increasing amounts of Flag-FBXL8 plasmids for 48 h, then the WB assay was performed to measure the protein levels. **(B)** Amino acid sequences of IκBα from the indicated species are highly conserved around the Y305 sites. **(C)** PANC-1 cells were transfected with Vector, WT or mutant HA-IκBα (Y305F) and Flag-FBXL8 plasmids for 48 h, then treated with MG132 (10 μM) for 4h. The co-IP assay was performed using anti-Flag antibody. **(D)** 293T cells were transfected with Vector, WT or mutant HA-IκBα (Y305F), Flag-FBXL8, and His-Ub K63 plasmids for 48 h, then the cells were lysed with ubiquitination lysis buffer followed by pull-down using Ni-NTA beads, and precipitates were analyzed by WB. **(E)** PANC-1 cells were transfected with HA-IκBα and Flag-FBXL8 plasmid in the presence or absence of the ABL1 inhibitor (Imatinib, 10 μM) for 48 h, then treated with MG132 (10 μM) for 4h. The co-IP assay was performed using anti-Flag antibody. **(F)** 293T cells were transfected with HA-IκBα, Flag-FBXL8, and His-Ub K63 plasmids in the presence or absence of the ABL1 inhibitor (Imatinib, 10 μM) for 48 h, then the cells were lysed with ubiquitination lysis buffer followed by pull-down using Ni-NTA beads, and precipitates were analyzed by WB. **(G, H)** PANC-1 and CFPAC cells were transfected with Vector, WT or mutant IκBα (Y305F) and Flag-FBXL8. Cell proliferation was measured using EDU assays (G). Transwell assays measured cell migration and invasion ability (H). Data are presented as the mean ± SEM; significance determined by one-way ANOVA with Tukey's multiple comparison (G, H). *p < 0.05, **p < 0.01, ***p < 0.001, ****p < 0.0001.

Phosphorylation of serine, threonine, and tyrosine residues in substrates often promotes recognition by F-box proteins^22^, ^23^. To explore whether IκBα is phosphorylated prior to binding with FBXL8, we used GPS6.0 to predict potential phosphorylation sites in IκBα (Fig. S4A). Combined with previous reports^24^-^27^, plasmids encoding IκBα mutants at these sites (S32A, S36A, Y42F, S283A, S288A, Y289F, T291A, S293A, T296A, T299A, Y305F) were constructed and co-expressed with FBXL8 to assess whether FBXL8 affects their stability (Fig. S4A). Notably, only the IκBα Y305F mutation remarkably reduced FBXL8-mediated stability regulation (Fig. 5A, S4B). Intriguingly, IκBα Y305 is evolutionarily conserved across species (Fig. 5B). Y305 phosphorylation occurs under steady-state conditions and suppresses the interaction between IKK and IκBα, thereby prevents the unwanted phosphorylation of the β-TrCP degron motif at the N-terminal (S32/S36) of IκBα and promotes the accumulation of IκBα^27^, ^28^. Consistent with this finding, Y305F blocked the phosphorylation of Y305 and enhanced the binding of IKK and β-TrCP to IκBα, and significantly reduced the interaction between FBXL8 and IκBα (Fig. 5C, S4C), suggesting that FBXL8 preferentially binds to IκBα when Y305 is phosphorylated, a state accompanied by dephosphorylation of the N-terminal S32/S36 residues. In addition, FBXL8-mediated ubiquitination of Y305F IκBα was significantly attenuated compared to WT IκBα (Fig. 5D). We have confirmed earlier that deletion of the PEST domain of IκBα does not affect the FBXL8-IκBα interaction. The PEST domain contains 305-310 amino acid residues that mediate IκBα-IKK binding, thereby deletion of the PEST domain abolishes the binding between IKK and IκBα, S32/S36 phosphorylation, and Y305 phosphorylation (Fig. S4D). These results further demonstrated that FBXL8 binding to IκBα is dependent on S32/S36 dephosphorylation, and Y305 phosphorylation could facilitate this process. Previous studies reported that ABL1 phosphorylates IκBα at Y305^27^. Treatment with an ABL1 inhibitor (Imatinib) or ABL1 shRNA suppressed FBXL8-IκBα binding and FBXL8-mediated IκBα ubiquitination (Fig. 5E, F, S4E, F). Functionally, WT IκBα suppressed PC cells proliferation, invasion, and migration, whereas the Y305-mutated IκBα failed to exert these inhibitory effects (Fig. 5G, H). These results suggest that ABL1-mediated phosphorylation of IκBα at Y305 potently facilitates the dephosphorylation of IκBα at S32/S36, followed by FBXL8-IκBα interaction.

### Reduced FBXL8 activates NF-κB signaling via promoting p65 nuclear translocation

**Figure 6:**
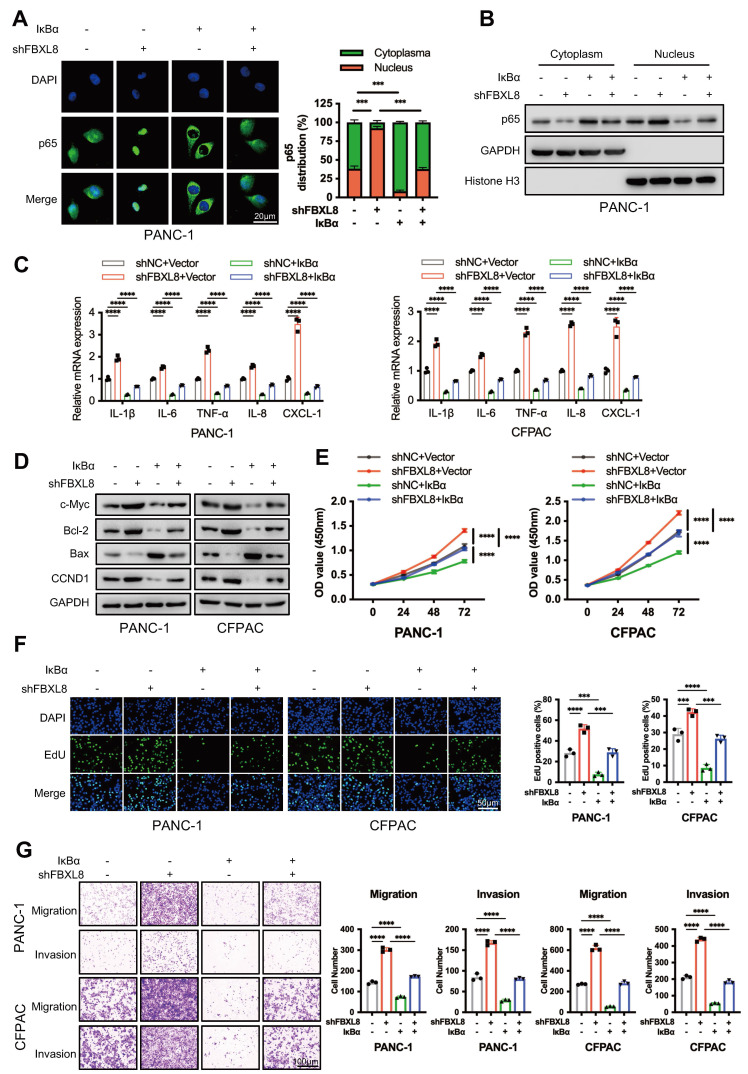
** FBXL8 stabilizes IκBα to inhibit p65 nuclear translocation and NF-κB signaling pathway. (A-G)** PC cells were transfected with shFBXL8 and IκBα plasmids for 48 h. p65 location was investigated using immunofluorescence staining (A). p65 protein levels in the cytoplasm and nucleus were measured by WB (B). The expression of p65 targeted genes were measured by qPCR (C) and WB (D). Cell proliferation was measured using CCK-8 (E) and EDU (F) assays. Transwell assays measured cell migration and invasion ability (G). Data are presented as the mean ± SEM; significance determined by one-way ANOVA with Tukey's multiple comparison (A, E-G) and two-way ANOVA with Bonferroni's multiple comparisons test (C). *p < 0.05, **p < 0.01, ***p < 0.001, ****p < 0.0001.

To determine whether FBXL8-mediated IκBα stabilization affects p65 nuclear translocation and NF-κB signaling, we knocked down FBXL8 alone or in combination with IκBα overexpression in PC cell lines. FBXL8 knockdown promoted p65 nuclear translocation, activated downstream proinflammatory cytokines and chemokines (IL-1β, IL-6, TNF-α, IL-8, and CXCL-1) and proliferation-related targets (c-Myc, Bcl-2, CCND1), inhibited Bax expression, and enhanced PC cells proliferation, invasion, and migration. Conversely, IκBα overexpression rescued p65 nuclear translocation and downstream molecule activation induced by FBXL8 knockdown, thereby suppressing cell proliferation, invasion, and migration (Fig. 6A-G). These results indicate that FBXL8 dysregulation-mediated IκBα reduction promotes PC progression by activating the NF-κB signaling pathway.

The activation of p65 will lead to the quick synthesis of new IκBα protein, the newly synthesized IκBα will be transported back to cytoplasm to bind p65 and quickly shut off the canonical NF-κB signaling^29^, ^30^. To address the potential impact of this classical feedback loop on FBXL8-mediated IκBα stabilization, we first examined the status of this feedback loop in PC cells. As shown in Fig. S5A, in two PC cell lines, TNF-α treatment led to a gradual and sustained decrease in IκBα protein levels over the time course, indicating impairment or loss of the classical IκBα autoregulatory loop in PC, consistent with previous findings in other tumor cells^31^. In contrast, 293T cells exhibited a complete and functional classical IκBα autoregulatory loop (Fig. S5A). Therefore, we used L-homopropargylglycine (HPG) labeling to distinguish newly synthesized and pre-existing IκBα pools in TNF-α-stimulated 293T cells. Western blot results showed that FBXL8 could maintain the stability of pre-existing IκBα regardless of whether new IκBα was synthesized (Fig. S5B).

**Figure 7:**
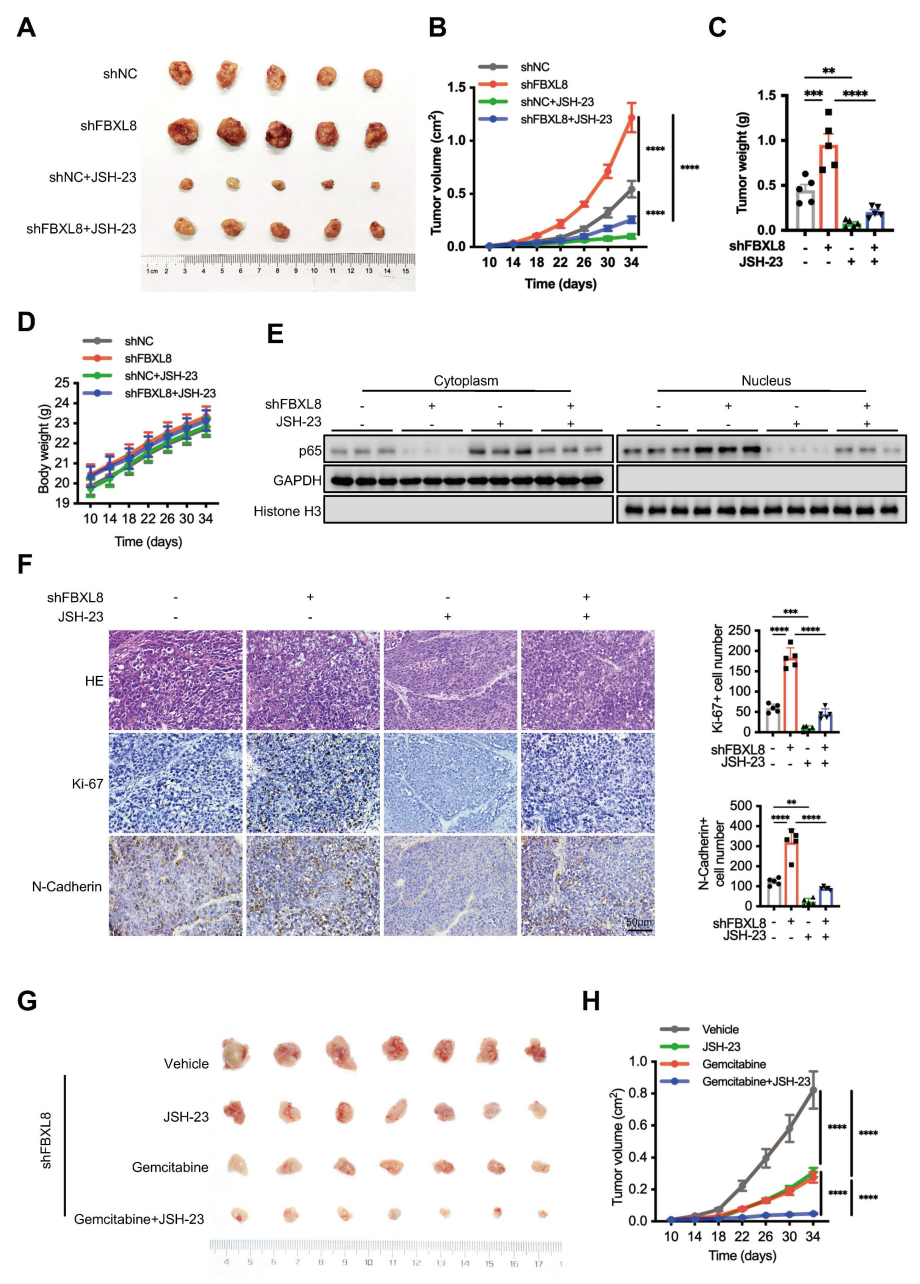
** The NF-κB inhibitor JSH-23 synergizes with gemcitabine to block FBXL8 low-expression-driven PC progression *in vivo*. (A-F)** Effects of FBXL8 knockdown and NF-κB inhibitor JSH-23 (20 mg/kg) on tumor growth in xenograft tumor model of PANC-1 cells. Macroscopic images were showed (A), and tumor size (B), tumor weight (C), and body weight (D) were determined. p65 protein levels in the cytoplasm and nucleus were measured by WB (E). Representative H&E staining and IHC staining of Ki-67 and N-Cadherin (F). (G, H) Effects of NF-κB inhibitor JSH-23 (20 mg/kg) and Gemcitabine (14.25 mg/kg) on tumor growth in xenograft tumor model of FBXL8 knockdown stable PANC-1 cells. Macroscopic images were showed (G), and tumor size (H) were determined. Data are presented as the mean ± SEM; significance determined by one-way ANOVA with Tukey's multiple comparison (B-D, F, H). *p < 0.05, **p < 0.01, ***p < 0.001, ****p < 0.0001.

JSH-23, a p65 nuclear translocation inhibitor, has been used in treating various tumors with NF-κB dysregulation^32^. We thus hypothesized that JSH-23 could serve as a therapeutic target for PC progression mediated by the FBXL8/IκBα/p65 axis. Treatment with JSH-23 suppressed PC cells proliferation, invasion, and migration induced by FBXL8 knockdown (Fig. S6A, B). Compared with controls, FBXL8 knockdown increased the size and weight of xenograft tumors in mice, whereas JSH-23 significantly blocked tumor growth *in vivo* without obvious systemic toxicity (Fig. 7A-D, S6C). FBXL8 knockdown led to p65 nuclear translocation, and a marked upregulation of the proliferation marker Ki-67 and EMT marker N-Cadherin, and this effect was significantly reversed by JSH-23 (Fig. 7E, F). Collectively, these data demonstrate that JSH-23 reverses FBXL8 dysregulation-induced PC progression *in vitro* and *in vivo*. Gemcitabine chemotherapy is used widely to treat human PDAC^33^, ^34^. To examine whether JSH-23 exerted a synergistic effect with gemcitabine, we used FBXL8 knockdown stable PANC-1 cells to establish mouse xenograft tumor models. The combination of gemcitabine and JSH-23 achieved better tumor control than either agent alone, indicating that gemcitabine plus JSH-23 could be a promising combination therapy for pancreatic cancer with low FBXL8 expression (Fig. 7G, H).

### NF-κB suppresses FBXL8 expression by activating YY1

**Figure 8:**
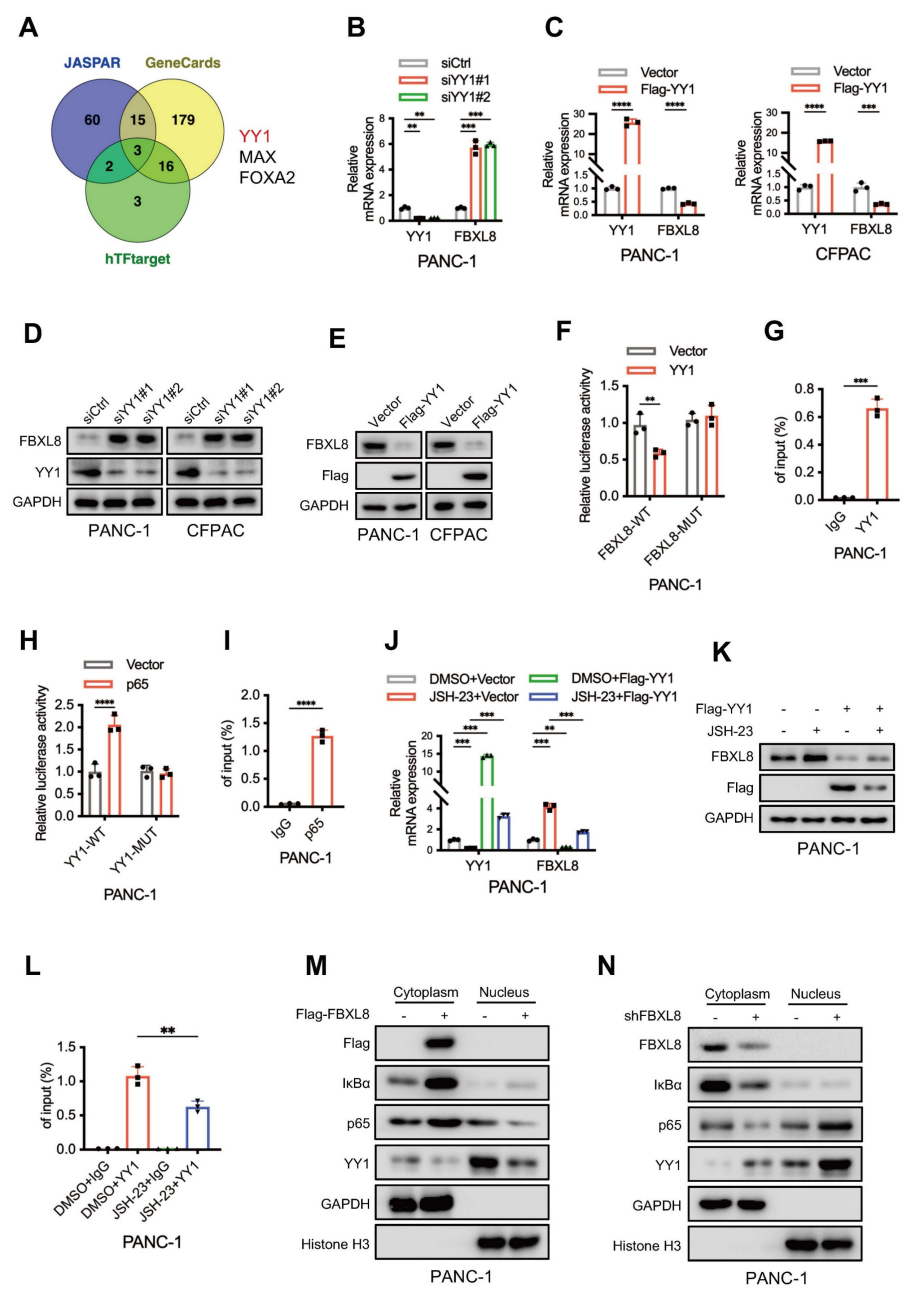
** NF-κB suppresses FBXL8 expression by activating YY1. (A)** Venn diagram showing potential upstream transcription factors of FBXL8 predicted by the hTFtarget, GeneCards, and JASPAR databases. **(B)** PANC-1 cells were transfected with nontargeting siRNA control (siCtrl) or siRNA targeting YY1 (siYY1) for 48 h. The expression of indicated genes was examined by qPCR. **(C)** PANC-1 and CFPAC cells were transfected with Vector or Flag-YY1 plasmids for 48 h. The expression of YY1 and FBXL8 was examined by qPCR. **(D)** PANC-1 and CFPAC cells were transfected with siCtrl or siYY1 for 48 h. The expression of YY1 and FBXL8 was examined by WB. **(E)** PANC-1 and CFPAC cells were transfected with Vector or Flag-YY1 plasmids for 48 h. The expression of YY1 and FBXL8 was examined by WB. **(F)** A luciferase reporter gene assay was performed in PANC-1 cells transfected with Vector or YY1 and WT-FBXL8 promoter (FBXL8-WT) or mutant-FBXL8 promoter (FBXL8-MUT) plasmids. **(G)** PANC-1 cells were used in the ChIP assay, and DNA-protein complexes were obtained and incubated with anti-IgG or anti-YY1 antibodies. Enriched DNAs were used for qPCR.** (H)** A luciferase reporter gene assay was performed in PANC-1 cells transfected with Vector or p65 and WT-YY1 promoter (YY1-WT) or mutant-YY1 promoter (YY1-MUT) plasmids. **(I)** PANC-1 cells were used in the ChIP assay, and DNA-protein complexes were obtained and incubated with anti-IgG or anti-p65 antibodies. Enriched DNAs were used for qPCR. **(J, K)** PANC-1 cells were transfected with Flag-YY1 plasmid and treated with NF-κB inhibitor JSH-23 (10 μM) for 48 h. The expression of YY1 and FBXL8 was examined by qPCR (J) and WB (K). **(L)** PANC-1 cells were treated with NF-κB inhibitor JSH-23 for 48h, and then used in the ChIP assay. DNA-protein complexes were obtained and incubated with anti-IgG or anti-YY1 antibodies. Enriched DNAs were used for qPCR.** (M, N)** PANC-1 cells were transfected with Flag-FBXL8 (M), or shFBXL8 (N) plasmids for 48 h. Indicated protein levels in the cytoplasm and nucleus were measured by WB. Data are presented as the mean ± SEM; significance determined by Student's *t*-test (C, F-I) and one-way ANOVA with Tukey's multiple comparison (B, J, L). *p < 0.05, **p < 0.01, ***p < 0.001, ****p < 0.0001.

To elucidate the underlying mechanism of FBXL8 downregulation in PC, we utilized three online databases (JASPAR, GeneCards, and hTFtarget) to predict potential upstream transcription factors of FBXL8. Three transcription factors, YY1, MAX, and FOXA2, were identified as candidates that might influence FBXL8 transcription (Fig. 8A). In PANC-1 cells, YY1 knockdown increased FBXL8 mRNA expression, whereas MAX and FOXA2 knockdown had no significant effect on FBXL8 expression (Fig. 8B, S7A, B). Conversely, YY1 overexpression decreased FBXL8 mRNA levels (Fig. 8C). YY1 also negatively regulated FBXL8 protein expression (Fig. 8D, E). Luciferase reporter assays showed that YY1 overexpression significantly reduced the luciferase activity of the WT FBXL8 promoter compared to the control, but had no significant effect on the activity of the mutant FBXL8 promoter (Fig. 8F). ChIP assays confirmed the binding of YY1 to the FBXL8 promoter (Fig. 8G, S7C). A previous study reported that NF-κB activates YY1 expression to promote PC progression and metastasis^35^. To further explore whether p65 directly binds to the YY1 promoter and regulates its expression, we first identified a conserved p65 consensus sequence (5'-GGGGCTTCCC-3') located in the YY1 promoter region (at positions -80 to -71 bp relative to the transcription start site). Luciferase reporter gene and ChIP assays confirmed the direct interaction between p65 and the YY1 promoter (Fig. 8H, I, S7D). Administration of the NF-κB inhibitor JSH-23 led to decreased YY1 expression and increased FBXL8 expression, yet this regulatory effect was significantly abolished upon YY1 overexpression (Fig. 8J, K, S7E, F). Moreover, the binding of YY1 to the FBXL8 promoter was attenuated in the presence of the NF-κB inhibitor (Fig. 8L, S7G). These data indicate that NF-κB suppresses FBXL8 expression by activating YY1. In addition, FBXL8 overexpression stabilized IκBα, suppressed p65 nuclear localization, and concurrently reduced YY1 expression; conversely, FBXL8 knockdown decreased IκBα, enhanced p65 nuclear accumulation, and increased YY1 expression, forming a feed-forward loop in which NF-κB-mediated YY1 upregulation inhibits FBXL8 transcription-further activating NF-κB (Fig. 8M, N).

### Low FBXL8 expression is associated with decreased IκBα expression and increased nuclear p65 in human PC tissues

**Figure 9:**
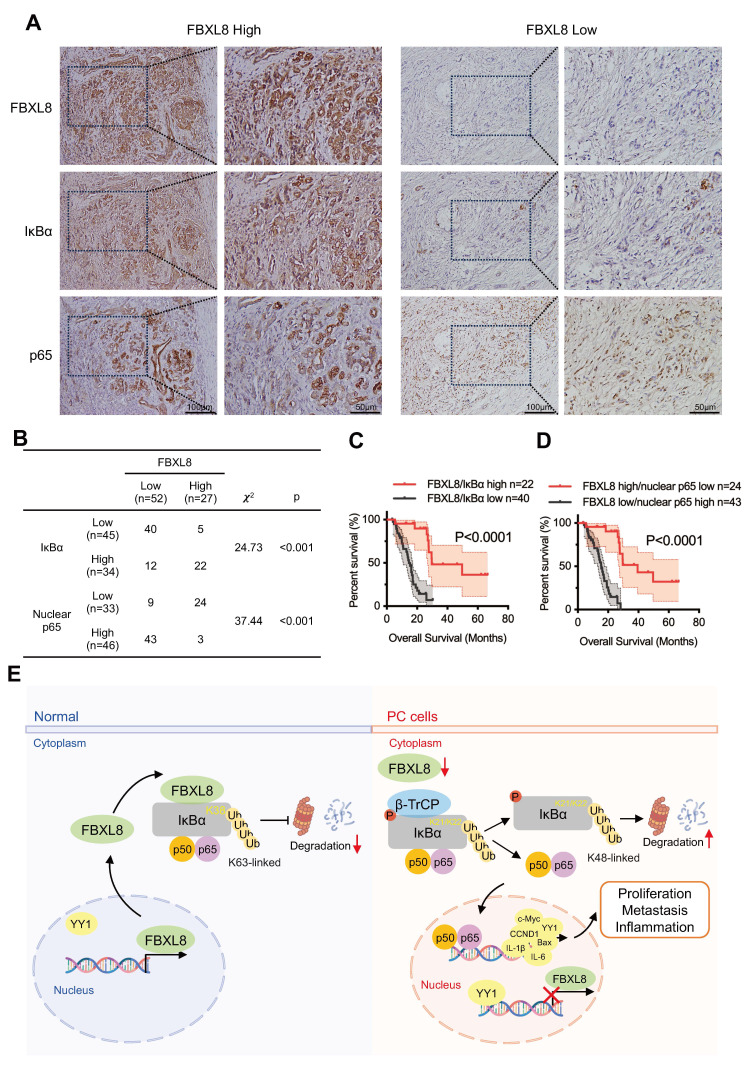
** Low FBXL8 expression is associated with decreased IκBα expression and increased nuclear p65 in human PC tissues. (A)** Representative IHC images showing the different expression of FBXL8, IκBα and p65 in PC tissues. **(B)** Correlation between FBXL8 and IκBα or FBXL8 and nuclear p65 in PC tissues (n=79). **(C)** The coexpression of low FBXL8 and IκBα showed a worse OS compared to these two proteins with high expression. **(D)** The expression of low FBXL8 and high nuclear p65 showed a worse OS compared to the expression of high FBXL8 and low nuclear p65. **(E)** Graphical summary of the role of FBXL8 imbalance in PC progression. In normal pancreatic cells, FBXL8 is highly expressed. It binds to dephosphorylated IκBα (S32/S36) and mediates K63-linked polyubiquitination at the K38 site of IκBα, thereby stabilizing IκBα and inhibiting the nuclear translocation of NF-κB p65. In PC cells, reduced FBXL8 weakens binding with IκBα. The phosphorylation of IκBα mainly occurring at S32/S36, which undergoes K48-linked polyubiquitination at the K21/K22 sites by β-TrCP. Such modification promotes IκBα degradation and enables p65 nucleus translocation. Nuclear p65 then upregulates the transcription of target genes including IL-1β, IL-6, CCND1, c-Myc, and Bcl-2, thereby driving PC proliferation and metastasis. Notably, this process also suppresses FBXL8 expression by activating YY1.

To assess the clinical correlation among FBXL8, IκBα, and p65 localization, we measured their expression in 79 PC patients and found a significant positive correlation between FBXL8 and IκBα expression, and a negative correlation between FBXL8 and p65 nuclear localization (Fig. 9A, B). Moreover, PC patients with concurrent high expression of FBXL8 and IκBα showed significantly better clinical outcomes than those with concurrent low expression (Fig. 9C). Patients with high FBXL8 expression and low p65 nuclear localization had significantly longer OS than those with low FBXL8 expression and high p65 nuclear localization (Fig. 9D). We also found that Ser32/36 phosphorylation, K48-linked polyubiquitination of IκBα, and YY1 levels were increased, while Y305 phosphorylation, K63-linked polyubiquitination and total protein levels of IκBα were reduced in PC tissues compared with adjacent non-tumor pancreatic tissues, consistent with the signaling pathways identified in our study and previous studies (Fig. S8A, B). These clinical observations align with *in vitro* findings in PC cell lines (PANC-1 and CFPAC): both cell lines exhibited higher Ser32/36 phosphorylation, K48-Ub of IκBα, and YY1 expression compared to the normal pancreatic duct epithelial cell line HPDE6-C7 (Fig. S8C, D). These results further support our mechanistic finding that FBXL8 stabilizes IκBα to inhibit NF-κB activation-with consistent clinical relevance in patient samples.

## Discussion

PC stands as one of the most malignant and lethal cancers, characterized by a grim prognosis with a five-year survival rate of less than 10%^36^. The cancer's high malignancy is due to its rapid growth and tendency to metastasize early to distant organs like the liver and peritoneum. Surgery, the only potentially curative option, is applicable to merely 10-20% of patients, mainly due to the advanced stage at diagnosis. Targeted therapies have emerged as a new treatment option. KRAS G12C inhibitors, such as sotorasib, can bring about certain efficacy in some patients, yet the remission duration remains short^37^, ^38^. PARP inhibitors like olaparib are applicable to patients with BRCA gene mutations, extending progression-free survival to a certain extent^39^. Anti-angiogenic drugs like bevacizumab, when combined with chemotherapy, can disrupt the tumor's blood supply^40^. However, these targeted drugs face limitations: their effectiveness is often short-lived, and the fibrotic microenvironment hinders drug delivery, making comprehensive, innovative treatment strategies urgently needed.

Ubiquitination is a PTM where ubiquitin (a 76-amino acid protein) is covalently attached to target proteins via its C-terminal glycine^41^. This process is mediated by sequential actions of E1 (ubiquitin-activating), E2 (ubiquitin-conjugating), and E3 (ubiquitin ligating) enzymes^42^, ^43^. While ubiquitination is often associated with proteasomal degradation, it also regulates diverse cellular processes through non-degradative mechanisms. Non-degradative ubiquitination provides a versatile mechanism for cells to modulate protein function, localization, and interaction networks^44^. By fine-tuning signaling transduction, autophagy, chromatin remodeling, cell cycle, and DNA repair, these modifications underpin cellular homeostasis and adaptive responses to stress^45^-^48^. Dysregulation of non-degradative ubiquitination has been linked to diseases like cancer, neurodegeneration, and inflammatory disorders, highlighting its therapeutic potential^49^-^51^. IκBα, a key negative regulator of the NF-κB signaling pathway, is canonically ubiquitinated and degraded to release p65 into the nucleus, thereby activating transcription of target genes^52^. Nevertheless, the role of non-degradative ubiquitination in regulating IκBα function remains unclear. In this study, FBXL8 binds to IκBα and mediates its ubiquitination, which in turn stabilizes the protein. Contrary to the conventional view that K48-linked ubiquitination mediates the degradation of IκBα, we found that K63-linked ubiquitination plays a critical role in stabilizing IκBα. Notably, we identified a previously unknown and evolutionarily conserved ubiquitination site, K38, of IκBα and confirmed that this site plays an essential role in FBXL8-mediated IκBα stabilization. Alternatively, K21/K22 of IκBα have been demonstrated to be ubiquitinated by β-TrCP, leading to IκBα degradation and NF-κB signaling activation^21^, ^53^.

FBXL8 is an F-box subunit of the SCF E3 ubiquitin ligase complex, recognizing substrates for ubiquitination. As a context-dependent regulator in tumors, it acts as a tumor suppressor in hematologic malignancies by promoting proteasomal degradation of cyclin D3 and c-Myc, inhibiting aberrant cell cycle progression and oncogenic transcription^54^, ^55^. Conversely, in breast cancer, FBXL8 facilitates degradation of tumor suppressors like CCND2 and IRF5, promoting cell survival, migration, and pro-tumorigenic cytokine production^56^. This dual role highlights its complex function in tumor biology, making it a potential biomarker and therapeutic target for precision cancer therapy. In our study, we for the first time revealed the tumor-inhibiting role of FBXL8 in PC. Through *in vitro* and *in vivo* assays, we found that FBXL8 suppress the proliferation, migration and invasion of PC cells. Mechanistically, FBXL8 induces K63-linked polyubiquitination of IκBα at K38 to stabilize the protein, thereby reducing p65 nuclear translocation and inhibiting NF-κB signaling activation. Consistently, we found that IκBα expression is positively correlated with FBXL8 expression in human PC and low level of FBXL8 correlates poor OS for PC patients, supporting our conclusion that FBXL8-IκBα axis has a critical role in regulating tumorigenesis. Given the critical role of inflammation in NF-κB activation and tumor progression^57^, ^58^, we explored whether inflammation acts as an upstream trigger and pathologically relevant driver of the FBXL8-NF-κB axis. We first focused on tumors with well-documented inflammatory backgrounds (including BRCA, LUAD, LUSC, PAAD, LIHC, and READ) and TCGA database analysis revealed consistent downregulation of FBXL8 in these inflammation-associated tumors (Fig. S9A). We further validated that TNF-α stimulation significantly inhibited FBXL8 expression in cell lines derived from these tumors; in addition, overexpressing FBXL8 in these cells led to a notable increase in IκBα expression (Fig. S9B, C). These findings collectively confirm that the FBXL8-NF-κB axis is not restricted to PC but is widely present in tumors where inflammation plays a critical pathological role.

Phosphorylation represents the predominant PTM governing substrate recognition and ubiquitination by F-box proteins^59^, ^60^. This modification typically creates phosphodegron motifs on target proteins, serving as specific recognition signals for F-box subunits within SCF (SKP1-CUL1-F-box) E3 ligase complexes. For instance, IKK-mediated phosphorylation of IκBα at Ser32/Ser36 and pervanadate-induced Tyr42 phosphorylation of IκBα triggers its ubiquitination and degradation to activate NF-κB^61^, ^62^. In anti-IgM-stimulated B cells, activated Btk phosphorylates IκBα at Tyr289/305 in the cytosol, promotes IκBα dissociation from the NF- κB complex occurs without its degradation, and drives p65 nuclear translocation to mediate early NF-κB target gene activation via BCR signaling^63^. In human embryonic kidney 293T cells and human osteosarcoma U2OS cells, the nuclear non-receptor tyrosine kinase c-Abl can enhance the stability of nuclear IκBα through Tyr305 phosphorylation, allowing IκBα to accumulate in the nucleus and thereby inhibiting NF-κB activation caused by TNF-α stimulation^27^. A recent study reported that phosphorylation of IRE1α prevents its binding with the SEL1L/HRD1 E3 ligase complex, suggesting that dephosphorylation facilitates the substrate recognition by E3 ligases^64^. Here, we demonstrated that the interaction between IκBα and FBXL8 is dependent on dephosphorylation of IκBα at S32/S36, and the phosphorylation of IκBα at Tyr305 obviously promotes the interaction. This suggests that phosphorylation of the Tyr305 residue does not lead to degradation of IκBα, but the specific mechanisms underlying its differential functions in distinct cell types still require further investigation.

## Conclusion

In conclusion, we revealed that loss of FBXL8 decreased the K63-linked polyubiquitination of IκBα and destabilizes it, leading NF-κB activation and PC progression. Specifically, we identify that FBXL8-mediated ubiquitination and stabilization of IκBα is dependent on K38 site. Moreover, NF-κB-mediated YY1 upregulation inhibits FBXL8 transcription to form a feed-forward loop (Fig. 9E). Therefore, the restoration of the FBXL8-IκBα axis may serve as a novel approach for PC therapy.

## Supplementary Material

Supplementary materials and methods, figures and tables.

## Abbreviations

UPS: ubiquitin-proteasome system
PC: pancreatic cancer
Co-IP: co-immunoprecipitation
MS: mass spectrometry
ChIP: chromatin immunoprecipitation
PDAC: pancreatic ductal adenocarcinoma
PTM: post-translation modification
IKK: IκB Kinase
CK2: Casein Kinase 2
β-TrCP: β-transducin repeat-containing protein
RPMI-1640: Roswell Park Memorial Institute-1640
FBS: fetal bovine serum
DMEM: Dulbecco's Modified Eagle's Medium
IMDM: Iscove's Modified Dulbecco's Medium
Ub: Ubiquitin
shRNA: short hairpin RNA
CDS: core coding region
qRT-PCR: quantitative real-time PCR
WB: Western blot
ECL: enhanced chemiluminescence
WCE: whole cell extraction
CHX: Cycloheximide
CCK-8: Cell Counting Kit-8
EdU: 5-ethynyl-2'-deoxyuridine
IHC: immunohistochemistry
ANOVA: one-way analysis of variance
OS: overall survival
TCGA: The Cancer Genome Atlas
ANT: adjacent non-tumor tissues
GEO: Gene Expression Omnibus
LRR: leucine-rich repeat
SRD: serin-rich domain
HPG: L-homopropargylglycine
