# Supplementary material for: FBXL8 Stabilizes IκBα and Negatively Regulated NF-κB Activation to Suppress Pancreatic Cancer Progression
Source: Int J Biol Sci. 2026 Jan 8;22(3):1461–79. doi: 10.7150/ijbs.122689 (PMC12839177; doi:10.7150/ijbs.122689)
Supplement: Supplementary file 1 — Supplementary materials and methods, figures and tables. [file ijbsv22p1461s1.pdf]

Supplementary materials and methods

Co-immunoprecipitation (co-IP), and Mass Spectrometry (MS)

Cells were lysed with Cell lysis buffer for Western and IP (P0013, Beyotime, China) supplemented with 1% protease inhibitor cocktail (Selleck, USA) on ice for 20 min. The supernatant was collected after centrifugation at 12,000 g for 10 min at 4°C. 10 percent of solution was used as the whole cell extraction (WCE). The remaining solution was incubated with 20 μL Protein A/G Plus-agarose beads (Santa Cruz, USA) for 10 min with rotation at 4°C to reduce non-specific binding. After centrifugation at 1,000 g for 5 min at 4°C, the supernatant was incubated with 5 μg primary antibody and 50 μL Protein A/G Plus-agarose beads overnight with rotation at 4°C. After washing with lysis buffer for 4 times, the precipitated proteins were eluted form beads with SDS-PAGE Sample Loading Buffer, and boiled for 10 min for WB and LC-MS/MS analysis by Applied Protein (Shanghai, China). To identify of FBXL8-interacting proteins and their ubiquitination sites, proteins were immunoprecipitated from FBXL8-overexpressing and control PANC-1 and CFPAC cells, followed by in-gel tryptic digestion to generate peptide fragments. Liquid chromatography (LC) separation of peptides from each sample was performed using an Easy nLC system (Thermo Scientific). The eluted peptides were subsequently analyzed with a Q-Exactive mass spectrometer (Thermo Scientific) operating in positive ion mode. Full-scan MS spectra (m/z 300-1800) were collected at a resolution of 70,000 at 100 m/z. Raw mass spectral data were processed using Proteome Discoverer Daemon (Thermo Scientific). Database searches were conducted against the UniProt human protein database with a maximum of two missed cleavages, and potential FBXL8 binding proteins (identified peptide segments > 1 in FBXL8 overexpressing cells) are presented in Tables S8 and S9. For the identification of ubiquitination sites on FBXL8-interacting molecules, ubiquitination-specific signatures (a characteristic diGly (glycine-glycine) remnant on modified lysine residues (resulting in a mass shift of 114.0429 Da) after trypsin cleavage) were analyzed via MS/MS, and the potential ubiquitination sites of FBXL8 binding proteins are shown in Table S10 and S11.

GST pull-down assay

HEK293T cells were transfected with WT or mutants HA-IκBα for 48 h, and then lysed in TNTE buffer (10 mM Tris HCl (pH 7.8), 150 mM NaCl, 1 mM EDTA, and 1.0% NP-40). Lysate was incubated at 4 °C for 3 h with 2 μg GST-FBXL8 LRR protein immobilized on Glutathione Sepharose 4B beads (GE Healthcare, UK). GST-FBXL8 LRR-bound complex was washed in TNTE buffer four times, boiled with SDS loading buffer, and analyzed by WB.

Cycloheximide (CHX) chase assays

After transfected for 48 h, cells were treated with CHX (100 μg/mL) to arrest protein synthesis. The protein samples at different time points were harvested, and analyzed by WB.

In vivo ubiquitination assays

After transfected for 48 h and treated with MG132 (10 μM) for 4 h, the cells were harvested, and 10 percent of solution was used as the WCE. The remaining solution was lysed in buffer A1 (6 M

guanidinium-HCl, 0.1 M Na_2HPO_4/NaH_2PO_4, 10 mM Tris-HCl (pH 8.0), 5 mM imidazole, and 10 mM β-mercaptoethanol) on ice for 30 min, and incubated with 50 μL Ni-NTA beads (Qiagen, Germany) overnight with rotation at 4°C. Then, beads were washed with buffer A (6 M guanidinium-HCl, 0.1 M Na_2HPO_4/NaH_2PO_4, 10 mM Tris-HCl (pH 8.0), 5 mM imidazole, and 10 mM β-mercaptoethanol), buffer B (8 M urea, 0.1 M Na_2HPO_4/NaH_2PO_4, 10 mM Tris-HCl (pH 8.0), and 10 mM β-mercaptoethanol), buffer C1 (8 M urea, 0.1 M Na_2HPO_4/NaH_2PO_4, 10 mM Tris-HCl (pH 6.3), 10 mM β-mercaptoethanol, and 0.2% Triton X-100), and buffer C2 (8 M urea, 0.1 M Na_2HPO_4/NaH_2PO_4, 10 mM Tris-HCl (pH 6.3), 10 mM β-mercaptoethanol, and 0.1% Triton X-100) once each time. The ubiquitinated proteins were eluted by buffer D (200 mM imidazole, 0.15 mM Tris-HCl (pH 6.7), 30% glycerol, 0.72 M β-mercaptoethanol, and 5% SDS), and boiled for 4 min for WB.

Cell Counting Kit-8 (CCK-8), 5-ethynyl-2'-deoxyuridine (EdU) and clone formation assays

CCK-8, EdU and clone formation assays were used to assess the cell proliferation. For CCK-8 assay, cells were seeded on 96-well plates at a density of 3,000 cells/well. After transfected, the OD450 was detected in next three days using CCK-8 reagent (Biomake, USA) according to the manufacturer's protocol. For EdU assays, transfected cells were seeded into 24-well plates at 50,000 cells/well. The next day, 50 mM EdU reagent was added and incubated 2 h. Then, cells were fixed with 4% paraformaldehyde (Biosharp, China), and permeabilized with 0.3% Triton X-100, followed by staining with BeyoClick™ EdU Cell Proliferation Kit with AF488 (C0071S, Beyotime, China), and images were captured with a fluorescence microscope (Olympus, Japan). For clone formation assay, transfected cells were seeded into 6-well plates at 1000 cells/well and cultured 14 days. The colonies were fixed with 4% paraformaldehyde for 15 min, and stained with 0.1% crystal violet (Solarbio, China) for 30 min. Then, the colonies were counted and photographed.

Cell migration and invasion assays

For cell migration, 500 μL DMEM/IMDM containing 10% FBS was added into the lower chamber. Then, the transwell chambers (Coring, USA) were placed, and 5×10^4 cells in 200 μL serum-free DMEM/IMDM were seeded into the upper chamber. After cultured for 24 h, cells were fixed with 4% paraformaldehyde for 15 min and stained with 0.1% crystal violet for 30 min. Cells which crossed chamber membrane was photographed by microscope and the cell numbers were quantified using ImageJ software. For cell invasion, the transwell chambers were precoated with 1:8 diluted Matrigel (Corning, USA) at 37°C for 3 h, and the rest procedures were the same as cell migration assay.

Immunohistochemistry (IHC)

Tissue specimens were fixed with 10% formalin for 48 h, and embedded with paraffin. Then, tissues were cut into 3-μm-thick sections, followed by deparaffinized, rehydrated, and antigen retrieval with citrate solution (P0081, Beyotime, China). After that, the DAB Detection Kit (Streptavidin-Biotin) (SP-9000, ZSGB-BIO, China) was used to stain the indicated protein according to the manufacturer's protocol, and hematoxylin (Biosharp, China) was used to counterstain. The IHC

score was calculated by multiplying the percentage of positive cells (0, <10%; 1, 10-25%; 2, 26-50%; 3, 51-75%; 4, 76-100%) and the intensity of staining (0, negative; 1, weakly positive; 2, moderately positive; 3, strongly positive). Samples with a score higher than 7 were high expression, while samples with a score less than or equal to 6 were low expression. The antibodies are presented in Table S5.

Immunofluorescence

Cells (1×10^4) were seeded in 35-mm confocal dishes (Nest, China). After washing with PBS, the cells were fixed with 4% paraformaldehyde for 15 min and blocked with 5% BSA for 1 h. The cells were incubated with the primary antibodies at 4 °C overnight. The next day, the cells were washed with PBS and incubated with Cy3/FITC-labeled goat anti-rabbit/mouse IgG (1:200, Beyotime, China) in dark for 1 h. Finally, Hoechst 33342 (Thermo Scientific, USA) was used to stain the nuclei. Immunofluorescence graphs were examined by confocal microscopy (Zeiss, Germany).

Bio-orthogonal noncanonical amino acid tagging (BONCAT)

Newly synthesized proteins were selectively enriched and analyzed using the BONCAT-based L-homopropargylglycine (HPG) Protein Synthesis Western Blot Kit (P1215S, Beyotime, China), strictly following the manufacturer's instructions. Briefly, transfected cells were cultured in methionine-free DMEM (Gibco, USA) supplemented with 1×HPG, a methionine analog that incorporates into nascent polypeptide chains during translation, and simultaneously treated with TNF-α (1 nM) and cycloheximide (CHX, 100 μg/mL) for the indicated durations. After treatment, cells were lysed, and HPG-labeled newly synthesized proteins were covalently conjugated to biotin via a copper-catalyzed click reaction. Biotin-tagged de novo synthesized proteins were then affinity-precipitated using Streptavidin Agarose (P2159, Beyotime, China), while pre-existing (non-newly synthesized) proteins remained in the supernatant. Both the precipitated fraction (newly synthesized proteins) and the supernatant fraction (existing proteins) were boiled with SDS loading buffer to denature proteins, and subsequent WB analysis was performed to detect the target proteins.

Luciferase reporter assay

The promoter sequence or the mutant sequence of human YY1 (GGGGCTTCCC→AAAATCCTTT) and FBXL8 (GCCATC→ATTGCT) was cloned into the pmirGLO vector. PANC-1 cells were seeded in 24-well plates and transfected with indicated plasmids. After 48 h, luciferase was measured using a Dual-Luciferase Assay Kit (Promega) according to the manufacturer's instructions.

Supplementary figures

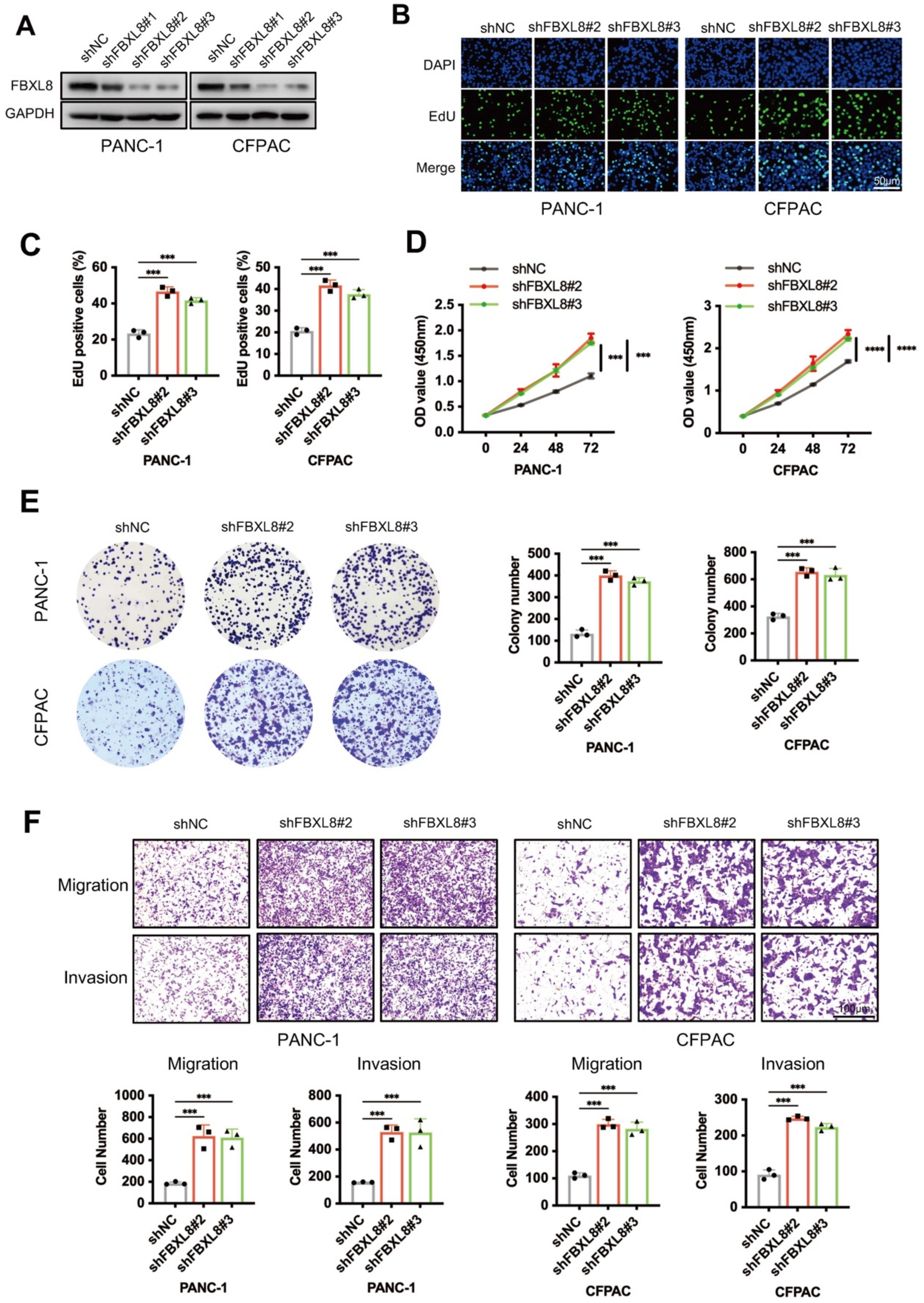

Figure S1. FBXL8 inhibits PC growth and metastasis.

(A-F) shFBXL8 was constructed in PANC-1 and CFPAC cells. The knockdown efficiency of shRNA targeting FBXL8 was detected by WB (A). Cell proliferation was measured by EDU (B, C), CCK-8 (D), and clone formation (E) assays. Transwell assays measured cell migration and invasion

ability (F). Data are presented as the mean ± SEM; significance determined by one-way ANOVA with Tukey's multiple comparison (C-F). *$p < 0.05$, **$p < 0.01$, ***$p < 0.001$, ****$p < 0.0001$.

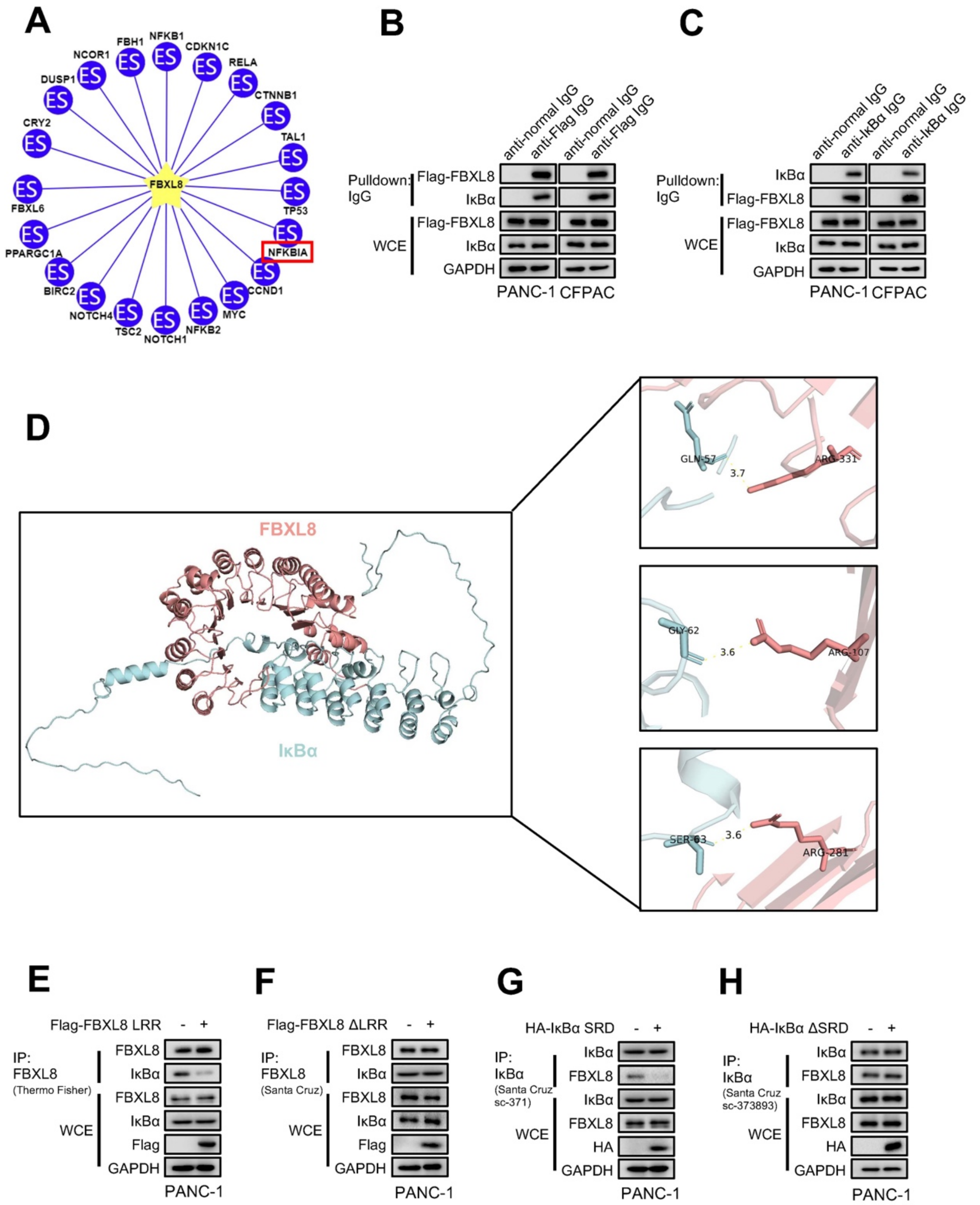

Figure S2. The LRR domain of FBXL8 interacts with the SRD of IκBα.

(A) Schematic diagram revealing the potential substrate of FBXL8 in the UbiBrower website. **(B, C)** PANC-1 and CFPAC cells were transfected with Flag-FBXL8 plasmid for 48 h. The co-IP assays were performed using anti-IgG or anti-Flag antibody (B) and anti-normal IgG or anti-IκBα antibody **(D)** Structural analysis using Pymol to visualize the potential combination sites of FBXL8 and IκBα. **(E)** PANC-1 cells were transfected with Flag-FBXL8 LRR plasmid for 48 h, then treated with MG132 (10 μM) for 4h. The co-IP assays were performed using anti-FBXL8 antibody (Thermo

Fisher, specifically raised against non-LRR regions). **(F)** PANC-1 cells were transfected with Flag-FBXL8 ΔLRR plasmid for 48 h, then treated with MG132 (10 μM) for 4h. The co-IP assays were performed using anti-FBXL8 antibody (Santa Cruz, specifically raised against LRR regions). **(G)** PANC-1 cells were transfected with HA-IκBα SRD plasmid for 48 h, then treated with MG132 (10 μM) for 4h. The co-IP assays were performed using anti-IκBα antibody (sc-371, Santa Cruz, specifically raised against non-SRD regions). **(H)** PANC-1 cells were transfected with HA-IκBα ΔSRD plasmid for 48 h, then treated with MG132 (10 μM) for 4h. The co-IP assays were performed using anti-IκBα antibody (sc-373893, Santa Cruz, specifically raised against SRD regions).

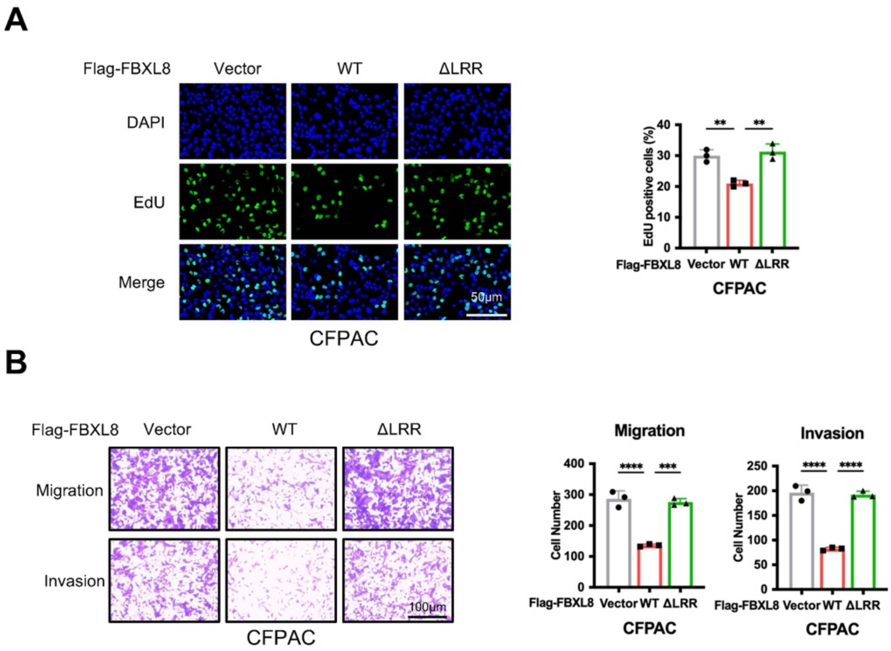

Figure S3. The deletion of the LRR domain abolished the inhibitory effect of FBXL8 on PC. **(A, B)** CFPAC cells were transfected with Vector, Flag-FBXL8 or Flag-FBXL8 ΔLRR. Cell proliferation was measured using EDU assays (A). Transwell assays measured cell migration and invasion ability (B). Data are presented as the mean ± SEM; significance determined by one-way ANOVA with Tukey's multiple comparison (A, B). *$p < 0.05$, **$p < 0.01$, ***$p < 0.001$, ****$p < 0.0001$.

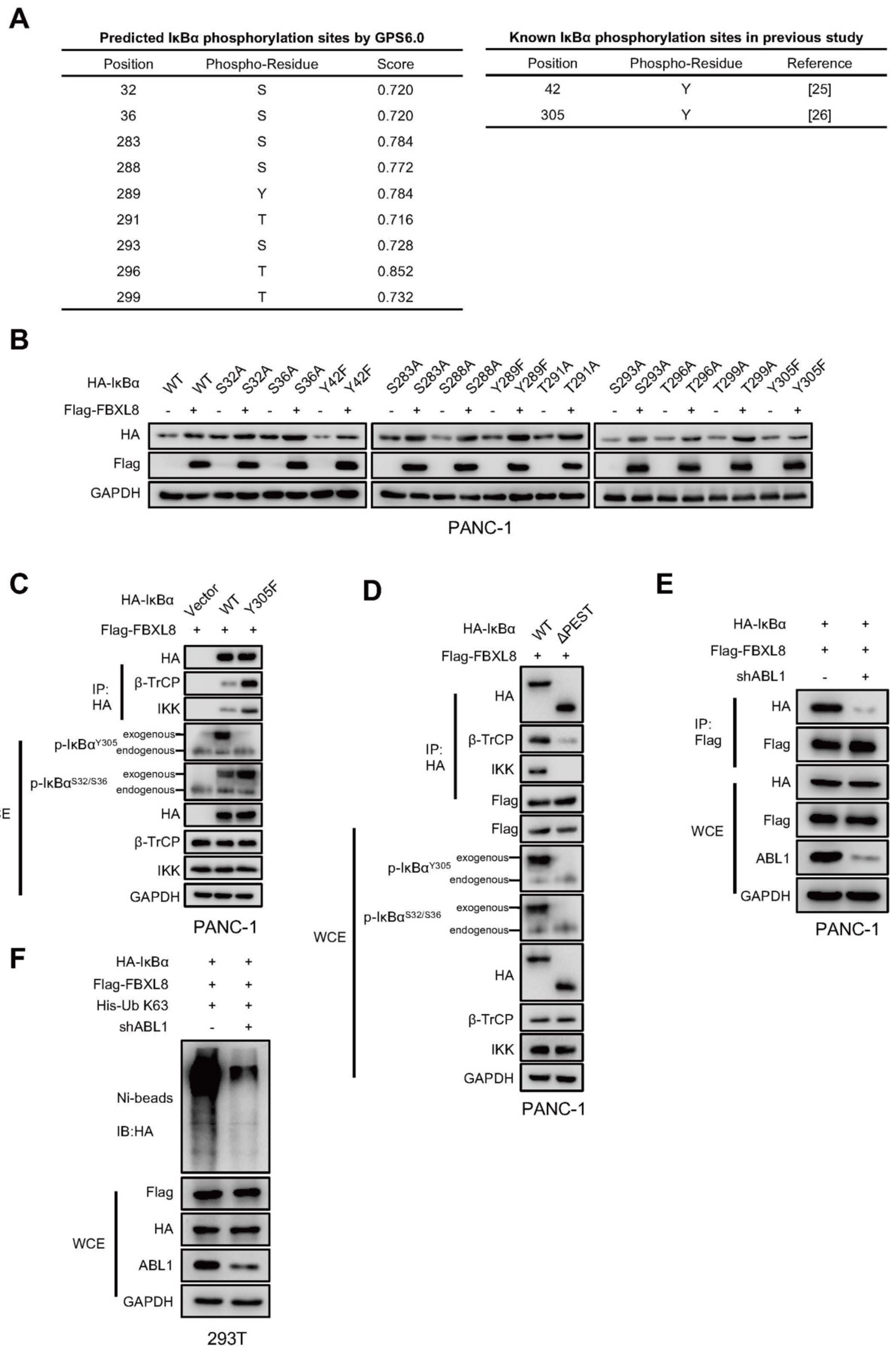

Figure S4. The binding of FBXL8 to IκBα is dependent on S32/S36 dephosphorylation, and Y305 phosphorylation can facilitate this process

(A) The potential phosphorylation sites of IκBα based on GPS6.0 software and previous reports. **(B)** PANC-1 cells were transfected with WT or mutant HA-IκBα (S32A, S36A, Y42F, S283A, S288A, Y289F, T291A, S293A, T296A, T299A, and Y305F) and Flag-FBXL8 plasmids for 48 h, then the

WB assay was performed to measure the protein levels. **(C)** PANC-1 cells were transfected with Vector, WT or mutant HA-IκBα (Y305F) and Flag-FBXL8 plasmids for 48 h, then treated with MG132 (10 μM) for 4h. The co-IP assay was performed using anti-HA antibody. **(D)** PANC-1 cells were transfected with HA-IκBα or HA-IκBα ΔPEST and Flag-FBXL8 plasmids for 48 h, then treated with MG132 (10 μM) for 4h. The co-IP assay was performed using anti-HA antibody. **(E)** PANC-1 cells were transfected with HA-IκBα and Flag-FBXL8 plasmid in the presence or absence of the shABL1 for 48 h, then treated with MG132 (10 μM) for 4h. The co-IP assay was performed using anti-Flag antibody. **(F)** 293T cells were transfected with HA-IκBα, Flag-FBXL8, and His-Ub K63 plasmids in the presence or absence of the shABL1 for 48 h, then the cells were lysed with ubiquitination lysis buffer followed by pull-down using Ni-NTA beads, and precipitates were analyzed by WB.

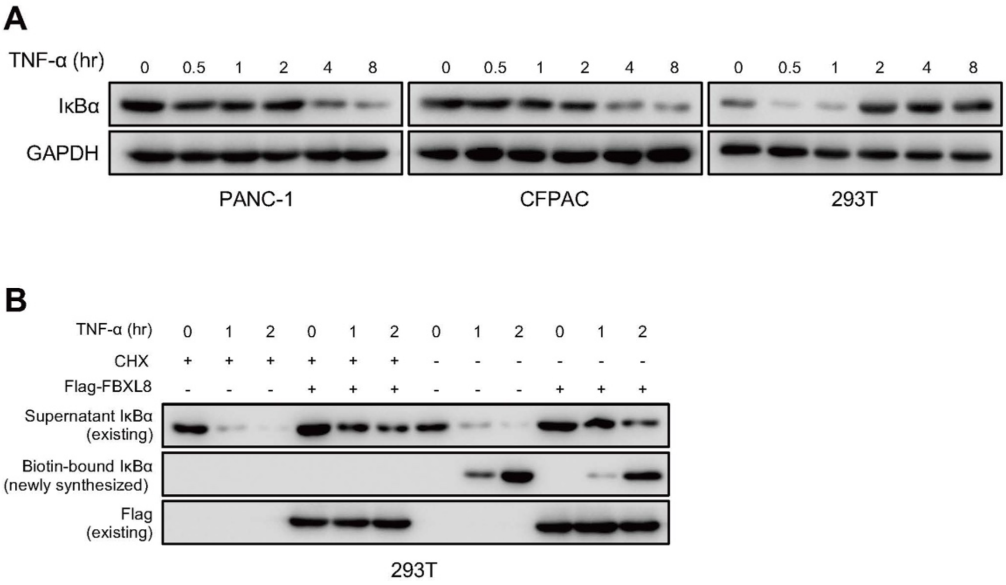

Figure S5. IκBα autoregulatory loop is absent in PC.

(A) PANC-1, CFPAC, and 293T cells were treated with TNF-α (1 nM) over 8 h time course. Attached cells were collected at each time point and proteins extracted for WB. **(B)** 293T cells were transfected with Vector or Flag-FBXL8, and then treated with TNF-α (1 nM) and CHX (100 μg/mL) for indicated times in methionine-free DMEM supplemented with 1× HPG. BONCAT based HPG-labeled newly synthesized proteins were precipitated using Streptavidin Agarose, while pre-existing proteins remained in the supernatant. WB was performed to detect the precipitated fraction (newly synthesized proteins) and the supernatant fraction (existing proteins) of the target proteins.

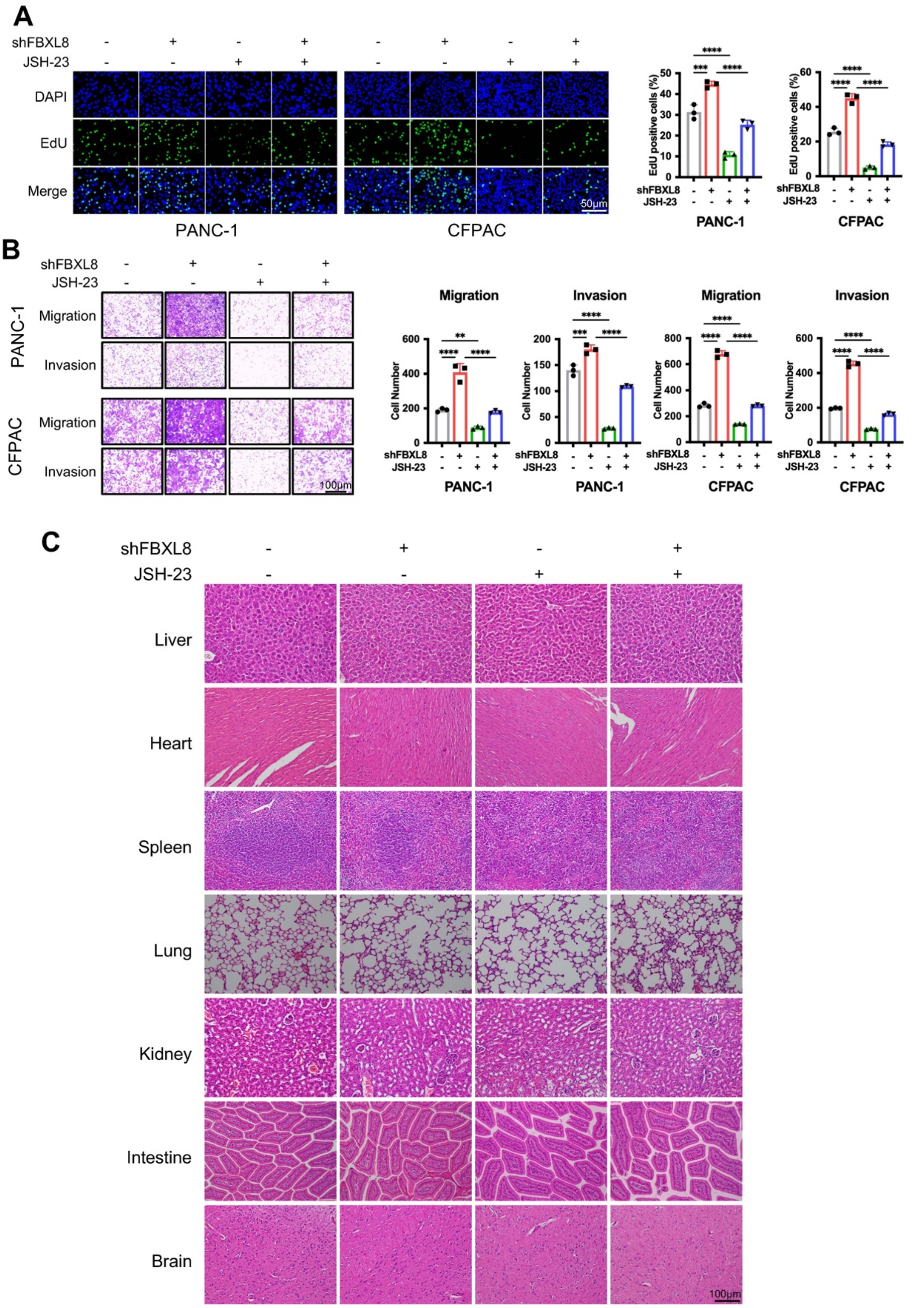

Figure S6. The NF-κB inhibitor JSH-23 blocks FBXL8 low-expression-driven PC progression in vitro.

(A, B) PC cells were transfected with shFBXL8 plasmid and treated with NF-κB inhibitor JSH-23 (10 μM) for 48 h. Cell proliferation was measured using EDU assays (A). Transwell assays measured cell migration and invasion ability (B). **(C)** Representative H&E staining of various

organs from xenograft tumor models. Data are presented as the mean ± SEM; significance determined by one-way ANOVA with Tukey's multiple comparison (A, B). *p < 0.05, **p < 0.01, ***p < 0.001, ****p < 0.0001.

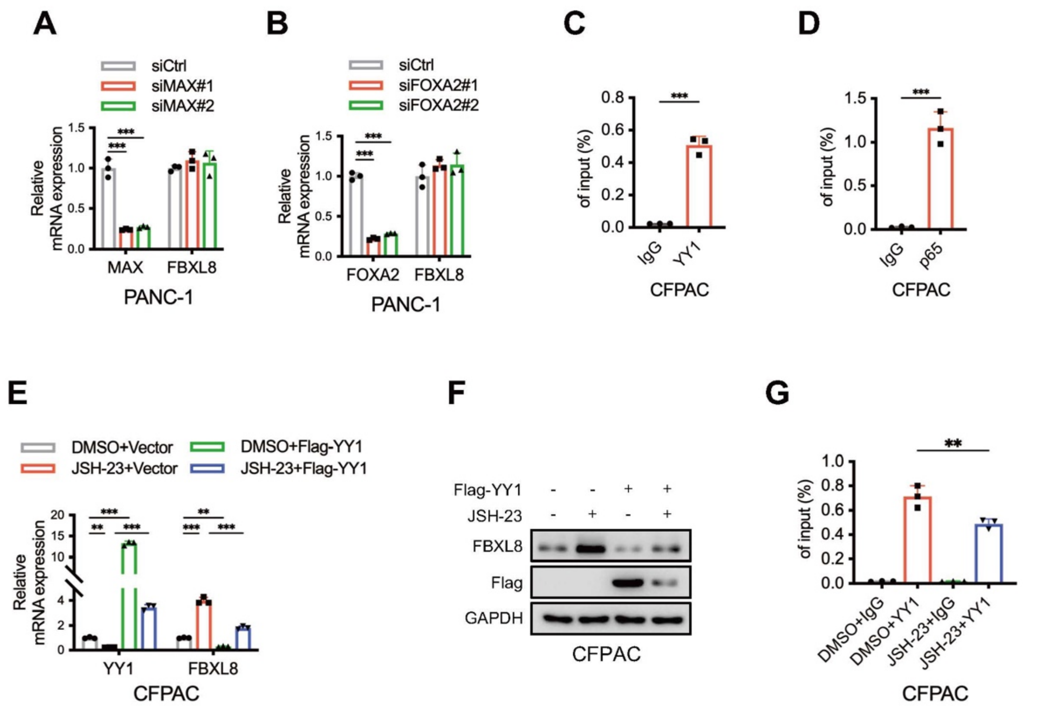

Figure S7. NF-κB suppresses FBXL8 expression by activating YY1.

(A, B) PANC-1 cells were transfected with nontargeting siRNA control (siCtrl) or siRNA targeting MAX (A) and FOXA2 (B) for 48 h. The expression of indicated genes was examined by qPCR. **(C)** CFPAC cells were used in the ChIP assay, and DNA-protein complexes were obtained and incubated with anti-IgG or anti-YY1 antibodies. Enriched DNAs were used for qPCR. **(D)** CFPAC cells were used in the ChIP assay, and DNA-protein complexes were obtained and incubated with anti-IgG or anti-p65 antibodies. Enriched DNAs were used for qPCR. **(E, F)** CFPAC cells were transfected with Flag-YY1 plasmid and treated with NF-κB inhibitor JSH-23 (10 μM) for 48 h. The expression of YY1 and FBXL8 was examined by qPCR (E) and WB (F). **(G)** CFPAC cells were treated with NF-κB inhibitor JSH-23 for 48h, and then used in the ChIP assay. DNA-protein complexes were obtained and incubated with anti-IgG or anti-YY1 antibodies. Enriched DNAs were used for qPCR. Data are presented as the mean ± SEM; significance determined by Student's t-test (C, D) and one-way ANOVA with Tukey's multiple comparison (A, B, E, G). *p < 0.05, **p < 0.01, ***p < 0.001, ****p < 0.0001.

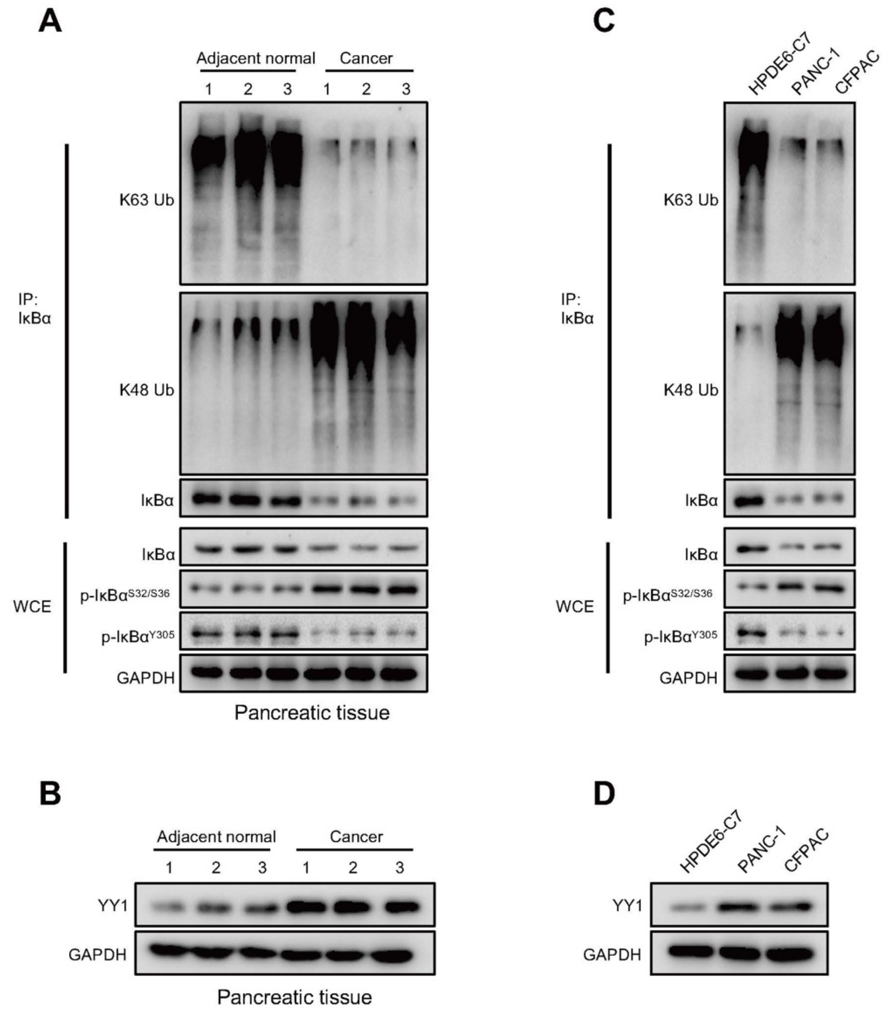

Figure S8. The phosphorylation, polyubiquitination of IκBα levels, and YY1 expression in PC. **(A)** The co-IP assays were performed using anti-IκBα antibody in PC and paired adjacent normal tissues. **(B)** The YY1 expression was measured in PC and paired adjacent normal tissues. **(C)** The co-IP assays were performed using anti-IκBα antibody in PC cell lines (PANC-1 and CFPAC) and normal pancreatic duct epithelial cell line HPDE6-C7. **(D)** The YY1 expression was measured in in PC cell lines (PANC-1 and CFPAC) and normal pancreatic duct epithelial cell line HPDE6-C7.

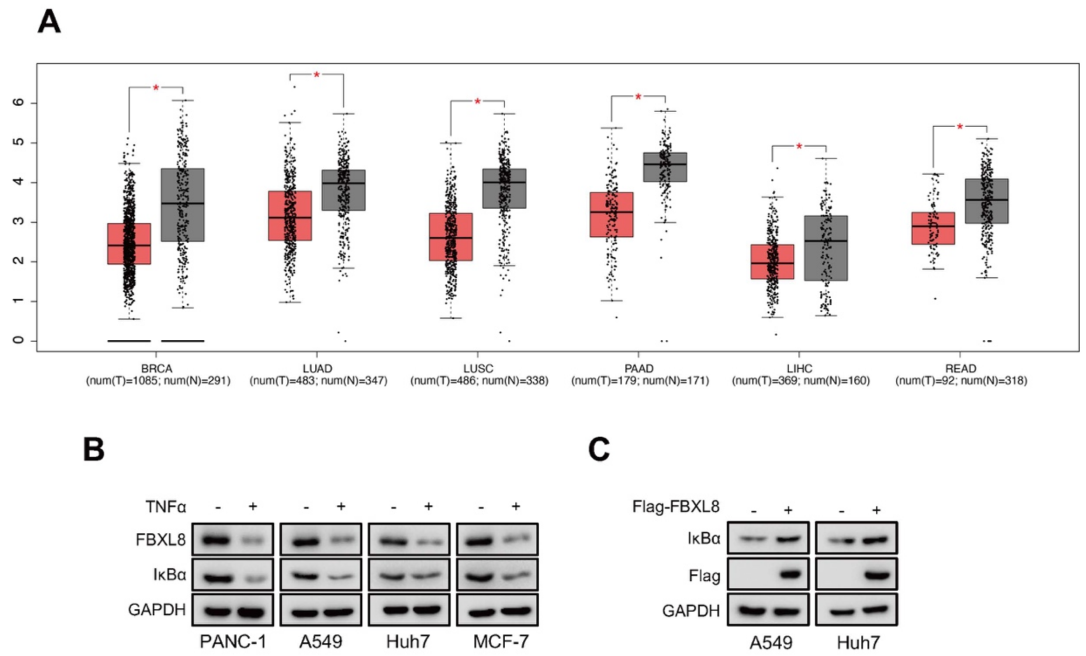

Figure S9. FBXL8-NF-κB axis widely exists in tumors where inflammation plays a critical pathological role.

(A) Relative FBXL8 mRNA levels in tumors (T) and ANT (N) across BRCA, LUAD, LUSC, PAAD, LIHC, and READ from TCGA and GTEx databases. **(B)** PANC-1, A549, Huh7, and MCF-7 cells were treated with TNF-α (1 nM) for 12 h, then cells were lysed for WB. **(C)** A549 and Huh7 cells were transfected with Vector or Flag-FBXL8, then cells were lysed for WB.

Supplementary tables

Table S1. siRNA sequences of target genes in this study.

Gene name	Direction	Sequence (5’-3’)
siCtrl	Sense	CAGCAGCAGUACGUUGAACTT
	Anti-sense	GUUCAACGUACUGCUGCUGTT
Human YY1#1	Sense	GACGACGACUACAUUGAACTT
	Anti-sense	GUUCAAUGUAGUCGUCGUCTT
Human YY1#2	Sense	CGAUGGUUGUAAUAAGAAGTT
	Anti-sense	CUUCUUAUUACAACCAUCGTT
Human MAX#1	Sense	CCACAGAAUAUAUCCAGUATT
	Anti-sense	UACUGGAUAUAUUCUGUGGTT
Human MAX#2	Sense	GCUCAUCAUAAUGCACUGGTT
	Anti-sense	CCAGUGCAUUAUGAUGAGCTT
Human FOXA2#1	Sense	GAACGGCAUGAACACGUACTT
	Anti-sense	GUACGUGUUCAUGCCGUUCTT
Human FOXA2#2	Sense	GCCCAUUAUGAACUCCUCUTT
	Anti-sense	AGAGGAGUUCAUAAUGGGCTT

Table S2. shRNA sequences of lentivirus for knockdown of target genes in this study.

Gene name	Sequence (5’-3’)
Negative control	UUCUCCGAACGUGUCACGU
Human FBXL8#1	UCUGGACAACAGUACCCUA
Human FBXL8#2	GAAGGCAUGCUGCCACCUU
Human FBXL8#3	AUCGAGCUGCUGAUGGUUC
Human ABL1	CCTTCATCCCTCTCATATCAA

Table S3. Antibodies used in this study.

Antibody	Source	Cat. #
FBXL8	Santa Cruz	sc-390582
FBXL8	Thermo Fisher	PA5-57325
GAPDH	Proteintech	10494-1-AP
Flag	Sigma	F1804-1MG
IκBα	Santa Cruz	sc-371
IκBα	Santa Cruz	sc-373893
HA	Roche	11867423001
GST	CST	2625
p-IκBα^{Y305}	ECM Biosciences	IX1045
p-IκBα$^{S32/S36}$	CST	9246
p65	CST	8242
Histone H3	CST	9715
c-Myc	CST	5605
Bax	CST	2772
Bcl-2	CST	4223
CCND1	Proteintech	60186-1-Ig
Ki-67	CST	9129
N-Cadherin	CST	13116
YY1	Proteintech	22156-1-AP
ABL1	Proteintech	32207-1-AP
β-TrCP	CST	4394
K63-linkage Specific Polyubiquitin	CST	5621
K48-linkage Specific Polyubiquitin	CST	8081
IKKα	CST	61294
Mouse IgG Isotype Control	CST	5145
Rabbit IgG secondary antibody	CST	7074
Mouse IgG secondary antibody	CST	7076
Rat IgG secondary antibody	CST	7077

Table S4. Primers for qPCR used in this study.

Gene	Primer direction	Primer sequence (5'-3')
FBXL8 (human)	Forward	ATCAGTTGCGAATGTGAGCTG
	Reverse	TCGAAGAGCGGTTTTTCTCCG
GAPDH (human)	Forward	CTGGGCTACACTGAGCACC
	Reverse	AAGTGGTCGTTGAGGGCAATG
IL-1β (human)	Forward	ATGATGGCTTATTACAGTGGCAA
	Reverse	GTCGGAGATTCGTAGCTGGA
IL-6 (human)	Forward	ACTCACCTCTTCAGAACGAATTG
	Reverse	CCATCTTTGGAAGGTTCAGGTTG
TNF-α (human)	Forward	GAGGCCAAGCCCTGGTATG
	Reverse	CGGGCCGATTGATCTCAGC
IL-8 (human)	Forward	ACTGAGAGTGATTGAGAGTGGAC

	Reverse	AACCCTCTGCACCCAGTTTTC
CXCL-1 (human)	Forward	GCTGGGATTCACCTCAAGAACATC
	Reverse	TGGGGACACCTTTTAGCATCTTCT

Table S5. Primers for ChIP-qPCR used in this study.

Gene	Primer direction	Primer sequence (5’-3’)
FBXL8 (human)	Forward	TGGTGGGCTAAGAGGAGTGT
	Reverse	GGGAGCCGAGACGAGCAG
YY1 (human)	Forward	TGGTGGGCTAAGAGGAGTGT
	Reverse	TTCCTCCCTCTGCCTT

Table S6. Correlations between FBXL8 with clinicopathological features in the 79 PC patients of the IHC cohort

Variable	FBXL8 low (n=52)	FBXL8 high (n=27)	χ^2	p
Gender				
Male	31	11	2.543	0.111
Female	21	16		
Age (years)				
＜60	29	9	3.584	0.058
≥60	23	18		
Tumor stage				
T1+T2	9	20	24.650	**＜0.001**
T3+T4	43	7		
Lymph node metastasis				
Negative	36	27	10.418	**0.001**
Positive	16	0		
Distant metastasis				
Negative	39	27	8.080	**0.004**
Positive	13	0		
Vascular invasion				
Negative	38	25	4.191	**0.041**
Positive	14	2		
Perineural Invasion				
Negative	35	23	2.910	0.088
Positive	17	4		
Differentiation degree				
Well	6	5	1.027	0.598
Moderate	40	18		
Poor	6	4		
TNM stage				
I+II	26	27	20.123	**＜0.001**
III+IV	26	0		
Histologic grade				
G1+G2	29	26	13.801	**＜0.001**
G3+G4	23	1		

Table S7. Univariate and multivariate analysis of different prognostic variables influencing overall survival in the 79 PC patients of the IHC cohort

Variable	Univariate			Multivariate	
	Cases	HR (95%CI)	p	HR (95%CI)	p
FBXL8 expression					
Low	52	0.13(0.06～0.29)	**＜0.001**	0.21(0.09～0.52)	**0.003**
High	27				
Gender					
Male	42	0.86(0.48～1.54)	0.61		
Female	37				
Age (years)					
＜60	38	0.99(0.56～1.74)	0.96		
≥60	41				
Tumor stage					
T1+T2	29	3.79(1.94～7.41)	**＜0.001**	2.34(1.09～5.02)	**0.049**
T3+T4	50				
Lymph node metastasis					
Negative	63	2.61(1.33～5.15)	**0.005**		0.71
Positive	16				
Distant metastasis					
Negative	66	3.02(1.44～6.32)	**0.003**		0.77
Positive	13				
Vascular invasion					
Negative	63	1.69(0.80～3.59)	0.17		
Positive	16				
Perineural invasion					
Negative	58	1.23(0.61～2.51)	0.56		
Positive	21				
Differentiation degree					
Well	11	1.31(0.79～2.15)	0.29		
Moderate	58	1.08(0.48～2.43)	0.86		
Poor	10				
TNM stage					
I+II	53	3.59(1.87～6.88)	**＜0.001**		0.35
III+IV	26				
Histologic grade					
G1+G2	55	3.16(1.64～6.10)	**0.001**		0.51
G3+G4	24				
Adjuvant chemotherapy					
Present	22	0.45(0.23～0.89)	**0.022**	0.36(0.16~0.80)	**0.012**
Absent	57				

Table S8. Summary of mass spectrometry analysis of FBXL8 interactors in PANC-1 cells.

Gene name	Protein ID	Unique peptide
FBXL8	Q96CD0	16
MCM3	Q53HJ4	8
TRIM21	P19474	7
EIF2S1	Q53XC0	5
RUVBL1	B5BUB1	4
QARS	P47897	4
UPF1	Q92900	3
RPS17	H0YN88	3
RPSA	C9J9K3	3
HEL-S-29	V9HWH2	3
PCBP2	Q15366	3
ANP32A	H0YN26	3
COPA	A0A3B3ISC6	3
G3BP2	Q9UN86	3
DBT	A0A7P0Z494	3
NFKBIA	A0A8V8TLC3	3
PHB	C9JZ20	3
PPIA	P62937	3
hCG_39985	Q53GB3	3
STAU1	B3KRE0	3
ARCN1	B0YIW5	3
ARPC3	Q2LE71	3
HSPA4	P34932	3
ATP5C1	B4DL14	3
MTHFD1	V9GZ78	3
SRSF1	J3KTL2	3
SMTN	P53814	3
PCBP1	Q53SS8	3
SFXN1	Q9H9B4	3
KCTD5	Q9NXV2	3
RCC2	Q9P258	2
MAGT1	A8MUP5	2
SEC22B	A0A087X1A9	2
AP2B1	B4E261	2
MYO6	A0A590UK71	2
HSPH1	B4DY72	2
C21orf33	H7C1F6	2
SET	A0A8I5KS71	2
SRSF4	A0A590UJK4	2
ETFA	A0A8I5KVC9	2
PSMA5	Q5U0A0	2
FLOT1	O75955	2

TRAP1	Q5CAQ4	2
AGPS	A0A7P0T8Q7	2
RPS29	A0A2R8Y851	2
DBN1	A0A2Z6ATB6	2
HNRNPH2	A0A384MDT2	2
DCTN2	A8K8J9	2
HNRNPH1	P31943	2
CSE1L	A0A384NKW7	2
PSMB3	P49720	2
ABCD3	B4DZ22	2
SLC25A24	A0A3B3IU96	2
CASC1	A0A6Q8PG26	2
NACA	F8VZJ2	2
KPNA2	A0A7I2V351	2
SF1	B4DX42	2
ADD1	E7EV99	2
MOV10	B7Z700	2
PPP1CB	P62140	2
PPP2R1A	A0A994J3N8	2
PGAM5	Q96HS1	2
LAMP2	B4E2S7	2
EIF3C	B4E2Z6	2
GLS	Q53TX0	2
LYN	B4DQ79	2
RFC5	A8K4Z2	2
TGM1	B4DRV1	2
IARS2	A8K5W7	2
FARSB	Q9BR63	2
XPOT	O43592	2
SNRPG	Q49AN9	2
RPS28	B2R4R9	2
BAG2	B3KM36	2
YWHAG	B4DE78	2
RUVBL2	B3KNL2	2
PSMD3	B3KNN7	2
PPP2CA	P67775	2
OTUB1	F5GYN4	2
PHF3	E7ER40	2
IDH2	B4DSZ6	2
TUBA1C	Q53GA7	2
NDUFS2	B7Z9L2	2
SPRR1A	B7ZLF8	2
SEC61A1	C9JXC6	2
EIF4E	Q32Q75	2

HSD17B11	D6RCD0	2
GCN1L1	Q92616	2
TCEB1	E5RHG8	2
FLOT2	Q6FG43	2
RAB1B	E9PLD0	2
RPL10	F8W7C6	2
HEL-S-156	V9HWH6	2
IPO5	H0Y8C6	2
HNRNPUL2-BSCL2	H3BQZ7	2
TXN	H9ZYJ2	2
MYO1D	J3QRN6	2
RPLP2	P05387	2
ASNS	P08243	2
SLC25A6	Q6I9V5	2
HEL-S-55	V9HW35	2
HEL-S-1	V9HWD6	2
STT3A	P46977	2
RANBP2	P49792	2
ACLY	Q4LE36	2
SNRPD3	P62318	2
ARHG	Q6ICQ8	2
SSRP1	Q08945	2
NSUN2	Q08J23	2
TWF1	Q12792	2
TUBB2A	Q13885	2
MCM6	Q14566	2
ITPR2	Q14571	2
VPS35	Q96QK1	2
NTPCR	Q5TDF0	2
LRRC47	Q8N1G4	2
KRT23	Q9C075	2
PAWR	Q96IZ0	2
IPO9	Q96P70	2
S100A14	Q9HCY8	2
YA61	Q9NZ23	2
GNG12	Q9UBI6	2
STOML2	Q9UJZ1	2
STRAP	Q9Y3F4	2

Table S9. Summary of mass spectrometry analysis of FBXL8 interactors in CFPAC cells.

Gene name	Protein ID	Unique peptide
NFKBIA	A0A8V8TLC3	10
KIF5B-ALK	V9HW29	3
HSPH1	B4DY72	2
SLC25A4	A8K787	2
DPM1	A0A0S2Z4Y5	2
GMPS	B4DUT7	2
AZGP1	P25311	2
CSRP1	Q9BTA4	2
ABCD3	B4DZ22	2
ANXA1	P04083	2
DNAJC7	Q99615	2
CASC1	A0A6Q8PG26	2
BPNT1	B7Z9J7	2
RTN4	Q53SY1	2
CDKN2A	A7LNE7	2
CROP	Q6PKC2	2
CDIPT	H3BUR9	2
CKM	B2R892	2
IPO7	B3KQG6	2
PPP2CA	P67775	2
PHF3	E7ER40	2
FEN1	B4DWZ4	2
ARL1	B4DZG7	2
U2AF2	B5BU25	2
RTN3	B7Z361	2
HEL32	B7Z9M9	2
ARHG	Q6ICQ8	2
DBF4B	Q8NFT6	2
ATAD3A	Q9NVI7	2
ATPIF1	Q9UII2	2

Table S10. Summary of ubiquitination sites within FBXL8 interactors identified by mass spectrometry in PANC-1 cells

Protein	Gene names	Sequence window	Positions
A0A8V8TLC3	NFKBIA	DRHDSGLDSMKDEEYEQMVKELQEIRLEPQE	38
A0A8V8TLC3	NFKBIA	DRHDSGLDSMKDEEYEQMVKELQEIRLEPQE	47
E7ER40	PHF3	TPDHLIRMSPEELASKELAAWRRRENRHTIE	290
B7Z9L2	NDUFS2	MELSGEMVRKCDPHIGLLHRGTEKL	10
P49792	RANBP2	KKTFEECQQNLMKLQKGHVSLAAELSKETNP	3049

Table S11. Summary of ubiquitination sites within FBXL8 interactors identified by mass spectrometry in CFPAC cells

Protein	Gene names	Sequence window	Positions
A0A8V8TLC3	NFKBIA	DRHDSGLDSMKDEEYEQMVKELQEIRLEPQE	38
A0A8V8TLC3	NFKBIA	DRHDSGLDSMKDEEYEQMVKELQEIRLEPQE	47
